# DentoMorph-LDMs: diffusion models based on novel adaptive 8-connected gum tissue and deciduous teeth loss for dental image augmentation

**DOI:** 10.1038/s41598-025-11955-2

**Published:** 2025-07-26

**Authors:** Hanaa Salem Marie, Mostafa Elbaz, Riham sobhy Soliman, Amira Abdelhafeez elkhatib

**Affiliations:** 1 Faculty of Artificial Intelligence, Delta University for Science and Technology, Gamasa, 35712 Egypt; 2 Department of Computer Science, Faculty of Computers and Informatics, Kafrelsheikh University, Kafrelsheikh, Egypt; 3 Lecturer of pediatric dentistry and dental public health, Faculty of Dentistry, Alexandria University, Alexandria, Egypt; 4 Lecturer of pediatric dentistry and dental public health, Faculty of Oral and Dental Medicine, KafrElsheikh University, Kafrelsheikh, Egypt

**Keywords:** Latent diffusion models, Dental image enhancement, Biologically-inspired algorithms, Oral disease detection, Pediatric dentistry, Medical image processing, Data processing, Machine learning

## Abstract

Pediatric dental image analysis faces critical challenges in disease detection due to missing or corrupted pixel regions and the unique developmental characteristics of deciduous teeth, with current Latent Diffusion Models (LDMs) failing to preserve anatomical integrity during reconstruction of pediatric oral structures. We developed two novel biologically-inspired loss functions integrated within LDMs specifically designed for pediatric dental imaging: Gum-Adaptive Pixel Imputation (GAPI) utilizing adaptive 8-connected pixel neighborhoods that mimic pediatric gum tissue adaptive behavior, and Deciduous Transition-Based Reconstruction (DTBR) incorporating developmental stage awareness based on primary teeth transition patterns observed in children aged 2–12 years. These algorithms guide the diffusion process toward developmentally appropriate reconstructions through specialized loss functions that preserve structural continuity of deciduous dentition and age-specific anatomical features crucial for accurate pediatric diagnosis. Experimental validation on 2,255 pediatric dental images across six conditions (caries, calculus, gingivitis, tooth discoloration, ulcers, and hypodontia) demonstrated superior image generation performance with Inception Score of 9.87, Fréchet Inception Distance of 4.21, Structural Similarity Index of 0.952, and Peak Signal-to-Noise Ratio of 34.76, significantly outperforming eleven competing diffusion models. Pediatric disease detection using enhanced datasets achieved statistically significant improvements across five detection models: +0.0694 in mean Average Precision [95% CI: 0.0608–0.0780], + 0.0606 in Precision [0.0523–0.0689], + 0.0736 in Recall [0.0651–0.0821], and + 0.0678 in F1-Score [0.0597–0.0759] (all *p* < 0.0001), enabling pediatric dentists to detect early-stage caries, developmental anomalies, and eruption disorders with unprecedented accuracy. This framework revolutionizes pediatric dental diagnosis by providing pediatric dentists with AI-enhanced imaging tools that account for the unique biological characteristics of developing dentition, significantly improving early detection of oral diseases in children and establishing a foundation for age-specific dental AI applications that enhance clinical decision-making in pediatric dental practice.

## Introduction

Pediatric oral disease detection represents one of the most critical and challenging aspects of children’s healthcare, with early childhood caries affecting over 60% of children globally and representing the most common chronic disease in pediatric populations^1^. Unlike adult dentistry, pediatric dental diagnosis must account for the rapidly evolving nature of deciduous dentition, where primary teeth exhibit distinct developmental patterns, accelerated disease progression, and unique vulnerability to pathological conditions^2^. The prevalence and types of dental problems in children differ dramatically from adults, with pediatric patients experiencing rapid caries progression that can advance from initial demineralization to pulpal involvement within months rather than years^3^. Early diagnosis and intervention in pediatric populations are particularly crucial because untreated dental diseases in children can lead to severe complications including pain, infection, nutritional deficits, speech development delays, and psychological impacts that extend far beyond oral health^4^. The unique characteristics of deciduous dentition—including smaller tooth size, thinner enamel (approximately 1 mm thick compared to 2.5 mm in permanent teeth), higher organic content, and different mineralization patterns—create diagnostic challenges that require specialized approaches fundamentally different from adult dental imaging and analysis.

In recent years, machine learning and deep learning have become powerful tools in dentistry for diagnosing oral diseases^5–7^. These technologies use large datasets of dental images, like X-rays and photos, to pinpoint patterns of various conditions^8,9^. Algorithms can be trained to accurately identify signs of issues such as caries and periodontal disease. By automating image analysis, these methods significantly boost diagnostic accuracy, cut down analysis time, and improve efficiency in dental practices^10^. Integrating artificial intelligence into dental practice not only streamlines diagnostics but also helps clinicians make more informed decisions based on predictive analytics, ultimately leading to better patient care for everyone.

Image augmentation is essential in preparing dental images for disease detection^11,12^. It increases the diversity of training data, improving the robustness of machine learning models^13^. Common techniques include rotation, scaling, cropping, and color adjustments. These methods enhance image quality and introduce useful variations. In dental imaging, where input quality directly affects model accuracy, augmentation helps create reliable systems that generalize across patients and imaging conditions^11–13^.

Latent Diffusion Models (LDMs) are effective for generating high-quality images and filling in missing data^14,15^. They work in a latent space, which makes image manipulation more efficient. LDMs learn complex patterns and produce realistic completions of missing regions. This improves dental image quality and preserves key features needed for diagnosis. In dental imaging, details like tooth shape and alignment are critical^16^. LDMs help maintain these features, even when parts of the image are damaged or unclear.

However, LDMs face limitations, particularly concerning the integrity between pixels and the number of missing pixels in augmented images. When significant portions of an image are missing or corrupted, maintaining the continuity of dental features can be challenging^17^. This can lead to inaccuracies in the detection of oral diseases, as the reconstructed images may not accurately reflect the true condition of the dental structures^11^. The potential for misdiagnosis underscores the need for more robust methods to fill these gaps effectively without compromising the integrity of the images. To address these challenges, we propose utilizing inspired metaheuristic algorithms as loss functions in our framework. By incorporating principles derived from pediatric dental development, these algorithms can enhance the imputation process, ensuring that the reconstructed images retain their original characteristics and are suitable for accurate disease detection.

Despite progress in dental image analysis, existing augmentation and reconstruction methods still lack biological awareness. Most models use generic loss functions that ignore the anatomical structure and developmental stages in dental images. This is especially problematic in pediatric cases, where dental features are still forming. Current methods fail to preserve critical details like gum structure and tooth transitions, which are key for accurate diagnosis.

To address this, we introduce a biologically inspired solution. We propose two novel loss functions GAPI and Deciduous Transition-Based Reconstruction (DTBR) based on gum tissue behavior and deciduous tooth development. These functions are integrated into Latent Diffusion Models to guide image reconstruction in a way that respects real anatomical structures. Our goal is to improve the realism and diagnostic value of dental image augmentation, especially in pediatric dentistry.

This approach aims to bridge the gap between the limitations of traditional LDMs and the requirements for high-quality dental image analysis.

This paper introduces a novel approach by drawing inspiration from two fundamental biological processes in pediatric oral development: the adaptive behavior of gum tissue and the developmental changes in deciduous teeth. These natural processes exhibit remarkable efficiency in maintaining structural integrity and facilitating transitions characteristics that can be harnessed computationally to enhance image processing techniques.

Our research proposes two innovative loss functions designed specifically for dental image enhancement. The first algorithm mimics the adaptive nature of gum tissue, which naturally maintains continuity and supports adjacent structures. By implementing an adaptive 8-connected pixel approach, we create a computational framework that preserves the integrity of dental images during the imputation process. The second loss function draws from the predictable yet dynamic patterns of deciduous teeth development, creating a mathematical model that optimizes pixel transitions in ways that reflect natural dental changes. Building upon these bioinspired algorithms, we demonstrate their effectiveness through comprehensive experimentation with various contemporary detection models. By augmenting dental image datasets using our proposed methods, we significantly improve the accuracy of oral disease detection systems, potentially enhancing clinical outcomes in pediatric dental care.

Contribution points

1. Development of two novel biologically-inspired metaheuristic algorithms: GAPI (Gum-Adaptive Pixel Imputation) that mimics gum tissue adaptive behaviors for image pixel imputation using an adaptive 8-connected pixel neighborhood evaluation system, and DTBR (Deciduous Transition-Based Reconstruction) that models deciduous teeth development patterns as mathematical loss functions with temporal context awareness for age-appropriate dental reconstructions.
2. Design of innovative loss functions that emulate intelligent gum tissue behavior and primary teeth developmental changes, maintaining image pixel integrity through adaptive neighborhood analysis while optimizing pixel imputation based on predictable yet dynamic patterns of deciduous dentition.
3. Integration of biologically-inspired approaches with Latent Diffusion Models (LDM) for enhanced dental image restoration and missing pixel imputation, creating a unified computational framework that combines traditional diffusion models with domain-specific biological knowledge.
4. Creation of an augmented dental image dataset using the proposed methods, improving training data quality for oral disease detection across six different dental conditions with pediatric-specific optimization tailored for developmental dental imaging challenges.
5. Validated the approach on multiple detection models, showing notable accuracy improvements and also an ablation step to prove the efficiency of each step in the hybrid methodology.

This study introduces several novel contributions to dental image processing. It presents the first application of gum tissue adaptive behavior as a computational model for pixel imputation and pioneers the use of deciduous tooth development patterns as mathematical loss functions. The work integrates biological processes from dental anatomy with machine learning techniques for image enhancement. A key innovation is the introduction of an adaptive 8-connected pixel approach that preserves spatial relationships in dental images. Unlike generic methods, the proposed loss functions are specifically tailored to address the unique challenges of dental imaging. By transferring biological knowledge from pediatric dentistry into computational vision models, the methodology bridges a critical gap. Overall, this cross-disciplinary approach offers a new pathway for combining dental expertise with advanced image reconstruction to support more accurate clinical outcomes.

The organization of the paper are as the follow; Sect. 2 presents the background and motivation; Sect. 3 presents the Literature Review; Sect. 4 presents the material and methods; Sect. 5 presents the results; Sect. 6 presents the discussion and Sect. 7 presents the conclusion.

## Background and motivation

Recently, as an emerging 3d visual representation, the neural signed distance function (SDF) has garnered much interest. Compared with traditional voxel grids and triangle meshes, a neural SDF represents 3d geometry as a continuous function and allows for compact storage of detailed geometry. A neural distance representation network (DRN) maps a street space point to its signed distance value for the learned geometry.

Recent advancements in deep generative models have enabled substantial progress in medical and dental image augmentation. Traditional approaches, such as GANs, have been used for dental image synthesis and caries detection^18,19^, but these models often suffer from training instability and limited anatomical control. In contrast, Latent Diffusion Models (LDMs) have emerged as a powerful alternative for generating high-resolution images with improved structural coherence^20,21^. Applications of LDMs in medical imaging include synthetic MRI generation^22^, histopathological image reconstruction^23^, and limited exploration in oral radiography^24^. However, these methods typically rely on general-purpose loss functions and do not incorporate domain-specific anatomical knowledge.

In the context of dental imaging, very few works have explored biologically informed augmentation strategies. Most augmentation pipelines apply conventional transformations such as flipping, rotation, and contrast adjustment, which fail to restore missing information or enforce structural consistency. Unlike prior LDM-based models, our work introduces biologically inspired loss functions tailored to pediatric dental anatomy, capturing soft-tissue behavior (via GAPI) and developmental transitions (via DTBR). To the best of our knowledge, this is the first integration of pediatric dental biology into a diffusion-based generative framework for image augmentation and imputation.

## Literature review

The modern era of deep learning has garnered considerable interest from researchers and professionals in a variety of domains. One of these recognizably popular fields is oral health care analysis. Supported with a large variety of datasets, models, and methodologies, research employing a variety of algorithms such as CNNs and GANs has risen significantly^25^. Unsurprisingly, the cruel implications of dental pathologies motivated researchers and professionals toward equipping dentists with novel, inexpensive systems for precise analysis, tracking, and classification^26^. The treatment of oral diseases involves a workflow that is both time-consuming and prone to error. Thus, understandable efforts were put toward researching deep learning-based (DL-based) algorithms as dental image analyzers^27^. Since the emergence of convolutional neural networks (CNNs) for image segmentation, researchers utilized their pyramid-like structure for treating dental images^28^. These one-stop systems were utilized to empower dentists toward precise tooth identification. Furthermore, second-stage classifiers were blended with deep features for canine-specific dental age estimation, as well as for oral pathology identification, detection, and classification. As such, exciting efforts toward modeling datasets containing both 2D and 3D imaging approaches have broadened the use of such algorithms, which can provide support for systems and mobile applications^29^. Parallel to these methods, deep learning architectures have shown exceptional potential in medical imaging and time-series analysis^30–33^. A psychosis-focused EEG dataset and introduces the Zipper Pattern (ZPat) feature extractor^34^. a novel channel-based transformation (TTPat) combined with Cumulative Weight–based Iterative Neighborhood Component Analysis (CWINCA), followed by classification using a t-algorithm k‑NN. Impressively, this model achieved classification accuracies exceeding 98.5% on EEG artifact detection tasks^35^.

These algorithms offer the promising potential of empowering dentists at multiple steps, such as tooth identification, detection of oral pathologies, error reduction, and clinical workflow streamlining. Besides giving insights to transform raw images into 3d digital impressions, localizing tooth surfaces via 3D bounding boxes and aligning panoramic radiographs with laterals were attempted, among others. This, ultimately, increases efficiency and improves treatment outcomes. Nevertheless, challenges arise in bringing these algorithms closer to clinical workflows. Datasets comprise a small amount of data; thus, by default, models are prone to over-detection. Those attempts utilized small pre-trained elements, thus restricting CNNs from conducting lab-independent analysis^36,37^. The performance of some DL-based algorithms remained unverifiable due to commercial intent. Hence, it remains infinitely valuable for the research community to compose a thorough review of articles focusing on deep learning (DL)-based methodologies applied to dental image segmentation. These publications will be classified based on their objectives and evaluated metrics. This review will pick the most prominent contenders of either side and investigate issues that, thus far, have stalled the advancement of such models.

### Current techniques in dental image processing

The exponential growth of imaging sensors and the accessibility of computational resources have increased the interest in different types and modalities of images for detection and recognition tasks across the medical, biometrics, and remote sensing fields. In society, images have become increasingly accessible; the medical imaging field has also benefited from larger archives made available by medical institutions. However, medical imaging frames differ from natural images due to intrinsic and extrinsic degradation factors. Most medical images have a lower pixel resolution (HR), higher noise levels, and a higher tendency to suffer from artifacts^38^. Consequently, problems arise in acceptable viewing and interpretation and the thwarting of associated analysis and recognition processes. Several fully automatic techniques require an image before performing interpretation and classification. However, images from lower-quality imaging sensors severely hinder these approaches’ performance. Before using an analysis- or recognition-based technique, it is often mandatory to enhance the quality of the acquired low-quality image.

Imposing a lens with a shorter focal length, increasing the illumination intensity, acquiring images of enough exposure time, or removing light pollution and haze can enhance image acquisition in a certain scene. However, such direct sensor-level enhancement is not possible in either the medical or remote sensing image acquisition system fields. As a result, in these fields, post-acquisition image enhancement methods are often applied. In practice, this is an especially challenging topic due to significant differences in image formation mechanisms across domains/fields, including sensor types, noise levels/characteristics, artifacts, and distortions. Nevertheless, in some fields, bioinspired approaches have been applied to design post-acquisition processors. Indeed, recent advancements in neurobiologically inspired and standardization basic designs of focal plane processing standards-inspired by the retina and feed-forward human visual systems, have shown promising preprocessing performance across multiple sensor domains.

This study presents a generic post-acquisition dental image enhancement framework inspired by the mammalian ocular anatomy, specifically the amphibian and the mammals eye, designed to complement existing odontological processors. The proposed framework is designed based on an image formation model of dental imaging involving the geometry of the tooth and the imaging sensor. Specifically, it aims to minimize the distance between the radiographic tooth shape and the grey-level tooth shape and hence enhance the input image(s) by matching them to the model output(s). The proposed image enhancement system has been evaluated via extensive experiments using a baseline dental image enhancement processor along with widely used vision and biomedical static image enhancement methods across several datasets.

### Diffusion models in image enhancement

Diffusion models (DMs) have been introduced for image enhancement in a fully data-driven fashion through the training of deep neural networks^39,40^. They have gained popularity due to their ability to generate high-fidelity images. These models learn the estimate of the data distribution as a Markovian process and do so by modeling a forward diffusion process defined as an Ornstein-Uhlenbeck process. The means and covariances (referred to collectively as parameters) of the Gaussian transition kernels are learned from the data distribution. A broader class of DMs is proposed based on Lévy motion. These Lévy DMs significantly expand the design space and facilitate sampling with a broader sampling range and higher sampling frequencies. Such mechanisms promote broad applications of the proposed models, with effective image synthesis, restoration and inpainting as demonstrations of the DMs’ potential.

The target object and embedding space of Lévy DMs can also be almost arbitrary, enabling them to leverage any domain-specific knowledge while maintaining data-driven learning^41^. Beyond the 2D image data type, 3D objects or even higher-dimensional video data can be used as input, and Lévy DMs can be defined over any underlying space structure, such as Euclidean space, manifold space, and graph space^42^. Such generality and versatility promise diverse and practical applications. The ever-evolving graph-based Lévy models are showcased to demonstrate the tool’s validity and effectiveness in processing irregular-data-based tasks. This framework can be adapted to other DMs, providing a powerful theoretical basis and practical reference for researchers and engineers in this domain.

### Loss functions in machine learning

Deep learning techniques rely on loss functions to measure model performance, optimize parameters, and implement the training process. The predominant approach to integrating loss functions into training is backpropagation^43^. This is a powerful gradient-based optimization technique that has transformed the way neural networks are trained. More specifically, differentiable models, such as neural networks, can be optimized by applying the chain rule of calculus to backpropagate gradients from the output layer to the input layer^44^. The weights in the model are adjusted utilizing the mini-batch stochastic gradient descent optimization algorithm. Quite recently, other optimization approaches have been developed for non-differentiable models, including natural evolution strategies, genetic algorithms, and particle swarm optimization.

Many applications require optimization of a custom loss function, defined in user code outside the model definition. For instance, in X-ray computed tomography (CT), some of the new applications for image processing or image reconstruction require a more complex loss function that uses more sophisticated metrics than mean square error (MSE)^45^. This strain is especially critical for reconstruction since reconstructions for different motions require different models to reconstruct the same data. These cannot be trained-to-convergence in the traditional sense since the multi-frame data will never be fully reconstructed with any single frame model. Instead, either to include multiple coarse-fine models in a deep learning framework or to progressively refine the model through a hierarchical optimization is implemented.

Moreover, strict penalization will lead to over-smooth reconstructions. Conversely, applying data consistency separately from the overall loss will yield noisy reconstructions at low complexity^46^. Hadamard reconstruction is favored to be integrated into optimized gradient descent, but this has suffered from null-space instabilities and failed to produce consistent quality by gradient descent^47^. Since the size of the objective space grows exponentially, the variety of local minima leads to irreproducible outputs. Thus, a next-generation regularization technique needs to tackle the challenges of customization for enabling new applications and extensive flexibility in the loss-penalty function.

### The related work

Diffusion models have shown significant promise in image restoration, providing a robust framework for enhancing low-light images and other challenging conditions. The wavelet-based conditional diffusion model (WCDM) enhances perceptual fidelity while reducing computational demands, making it suitable for dental applications where detail is crucial^48^. The framework’s ability to produce high-quality images can significantly aid dental diagnostics and treatment planning, enhancing the visualization of critical anatomical features. By integrating few-shot learning techniques, the model can adapt to diverse dental imaging scenarios, improving its utility in clinical settings^49^.

Diffusion models, particularly those based on U-Net architectures, have shown promise in image restoration tasks, enhancing detail preservation and reducing artifacts. Implementing loss functions that specifically address the characteristics of gum tissue and deciduous teeth can enhance model training, leading to better image quality and segmentation accuracy^50^. The framework can generate synthetic dental images, reducing the need for extensive annotated datasets, which are often difficult to obtain in medical imaging. The integration of dynamic imaging techniques, such as those used in Dynamic Cell Imaging (DCI), can facilitate high-resolution displays of dental tissues, aiding in segmentation and analysis^51^.

GANs have been shown to enhance medical images by addressing the limitations of traditional loss functions, which often fail to capture high-frequency details essential for clinical relevance^52^. Techniques such as fractional differential theory combined with wavelet decomposition have been proposed to enhance images across different domains, improving overall image quality and detail recovery^53^. Generative Adversarial Networks (GANs) hold significant promise for advancing dental radiology in clinical practice, education, and research. Dental radiographs are fundamental diagnostic tools that help assess a patient’s oral health, detect abnormalities, and guide treatment planning. However, acquiring large, diverse, and representative datasets of dental radiographs remains a challenge due to privacy concerns and limited access, especially when compared to widely available datasets such as ImageNet^54^, CheXpert^55^, or MIMIC-III^56^.

GANs offer an innovative solution by enabling the creation of realistic synthetic radiographs that can supplement existing datasets. This synthetic data can improve the training and validation of deep learning models used in dental image analysis, particularly when real-world data is scarce or imbalanced. In addition to data generation, GANs are also effective in tasks like data augmentation, image denoising, super-resolution, and domain adaptation—such as translating images between modalities like MRI and CT scans^57^. They can also support semantic segmentation by identifying anomalies at the pixel level, further enhancing diagnostic accuracy.

By addressing the limitations in data availability and quality, GANs have the potential to significantly enhance the capabilities of AI in dental radiology. A Review and Benchmarking of GAN-Based Approaches in Dental Imaging Literature is represented in Table 1. Table 2 represents various studies that have tackled the challenge of solving.

**Table 1:** Comparative evaluation of selected studies utilizing GANs in dental imaging.

References	Application	Model/Modality	Task	Dataset(s)	Performance
Hu et al.^58^.	Correction of Imaging Artifacts in Low-Dose Dental CT Scans	WGAN/ Low-dose dental CT	Artifact correction	Total: 44 patients (high/low-quality image pairs) Train: 15,246 pairs Validation: 3696 pairs Test: 5082 pairs	PSNR, SSIM: higher compared to traditional GAN and CNN Higher subjective scores considering noise suppression, artifact correction, detail restoration and comprehensive quality
Hegazy et al.^59^.	Image denoising by transfer learning	WGAN, U-WGAN, U-Net/ Medical CT (high/low dose), low dose dental CT	Denoising	Train: 5100 high/ low image pairs, fne tune with 3006 dental CT image pairs (from 2 skull phantoms) Test: 900 images	PSNR: 32.75 (U-Net) versus 27.42 (U-WGAN) versus 23.96 (WGAN) SSIM 0.974 (U-Net) versus − 0.962 (U-WGAN) versus 0.953 (WGAN)
Huang et al.^60^.	Cephalogram Synthesis and Landmark Detection from 2 cone-beam projections	Pix2pix CNN (LeNet-5, ResNet50)/Head CT (CQ500 head CT dataset), lateral cephalograms (ISBI dataset)	Domain transfer, landmark detection	Total: 491 CT scans Test: 5 CT scans	Average PSNR = 33.8 (Concerning the cephalograms synthesized from 3D CBCT volumes.) Pix2pixGAN also achieves best performance in super resolution (average PSNR = 32.5) Automatic landmark detection = 86.7% Successful detection rate in the 2 mm clinical acceptable range on the ISBI Test1data
Moran et al.^61^.	Super-Resolution GAN-Based Enhancement of Digital Periapical Radiographic Images	SRGAN, CNN (SRCNN)/ Periapical images	Super-resolution	Transfer learning: 102-fowers dataset (1792 images), pneumonia chest X-ray dataset (5856 images) Train: 228 images Test: 120 images	The results of SRGAN models using transfer learning were better on average for MSE (13.19 ± 6.31), PSNR (37.70 ± 2.96), SSIM (1.00 ± 0.00), and MOS (3.40 ± 0.55)
Park et al.^62^.	Forecasting 3D Facial Changes After Orthodontic Intervention	Conditional GAN-Based Generation of Post-Treatment CBCT Images from Pre-Treatment Data	Modeling and Forecasting Therapeutic Outcomes	Train 268 scan pairs Test 44 scan pairs (internal), 19 scan pairs (external)	Mean prediction error = 1.2 ± 1.01 mm Accuracy = 80.8% More than 50% of the experienced orthodontists were unable to distinguish between real and predicted images
Krishnamoorthy et al.^63^.	Automated Detection and Classification of TMJ Osteoarthritis in Panoramic Imaging	Deep Learning Pipeline with Optimized GAN and Faster R-CNN for Panoramic Dental Imaging	Generation, object detection	Total: 6000 images (augmented use optimized GAN) Train: 3900 images Test: 2100 images	Accuracy = 94.59% (Optimized GAN— faster RCNN)

**Table 2:** Comparative summary of related work and this Study.

Study	Dataset	Model Type	Metrics	Limitations	Novelty Gaps Filled
Shirsat et al.^64^	Private 2D dental X-rays	GAN (Pix2Pix)	SSIM, FID	Unstable training; low anatomical control	Dental image synthesis with GAN
Zhang et al.^65^	Pediatric panoramic images	U-Net + GAN	Accuracy, Precision	No pixel-level reconstruction	Caries detection
Wang et al.^66^	3D brain MRI (public)	InverseSR (LDM)	PSNR, SSIM	Non-dental domain	LDM for MRI super-resolution (github.com, reddit.com, reddit.com)
Grebe et al.^67^	Histopathology (artifact data)	LDM	SSIM, FID	Artifact restoration only, generic losses	Latent artifact removal
Ho et al.^68^	Histopathology FS→FFPE slides	LDM	FID, morphological metrics	Morphological fidelity not fully evaluated	Domain translation in histopathology
Zhang et al.^69^	H&E histopathology	LDM features + segmentation	Dice, IoU	No generative augmentation focus	Segmentation via LDM features
This paper	Pediatric dental images (custom)	LDM + Bioloss (GAPI + DTBR)	SSIM, FID, Precision, Recall, F1, mAP	Tested synthetic only; limited clinical validation	First biologically grounded LDM for pediatric dental imaging

##  Materials and methods

**Fig. 1:**
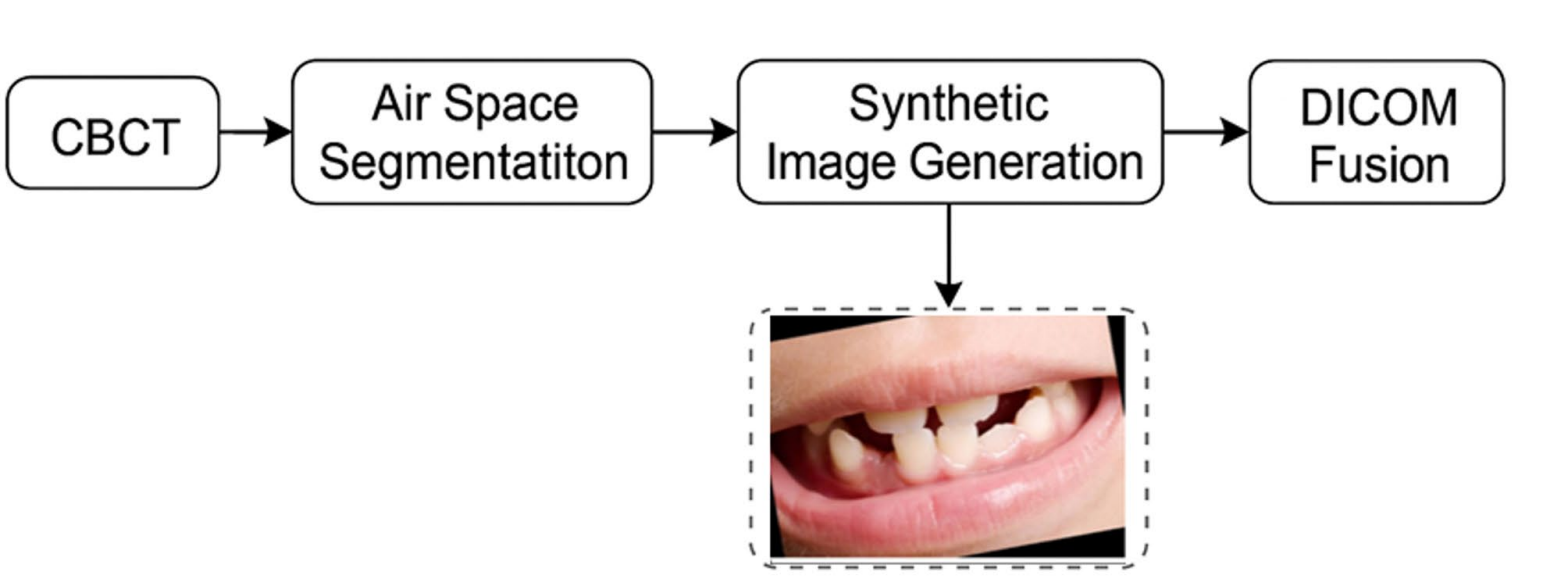
GAPI workflow.

**Fig. 2:**
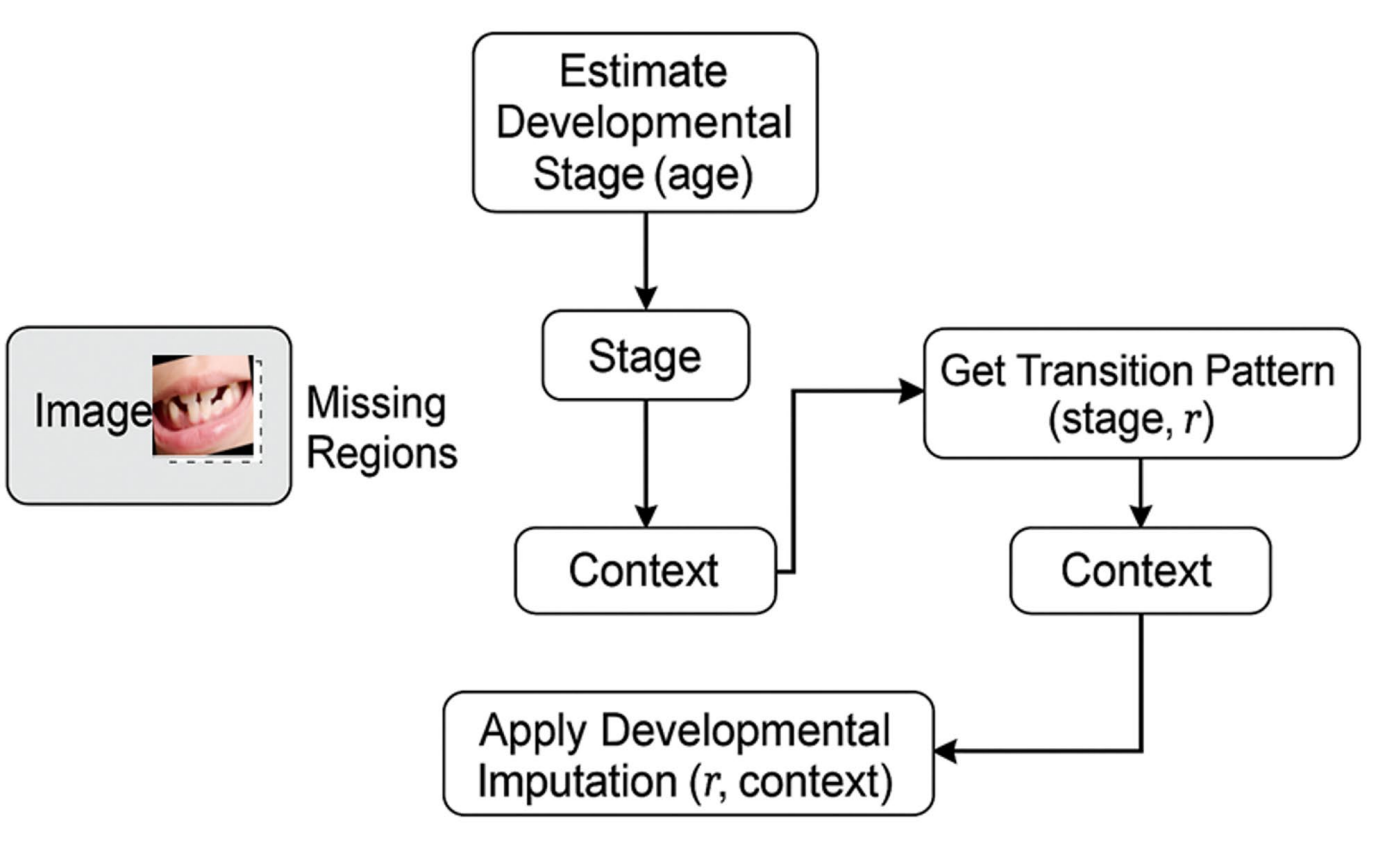
DTBR workflow diagram.

**Fig. 3:**
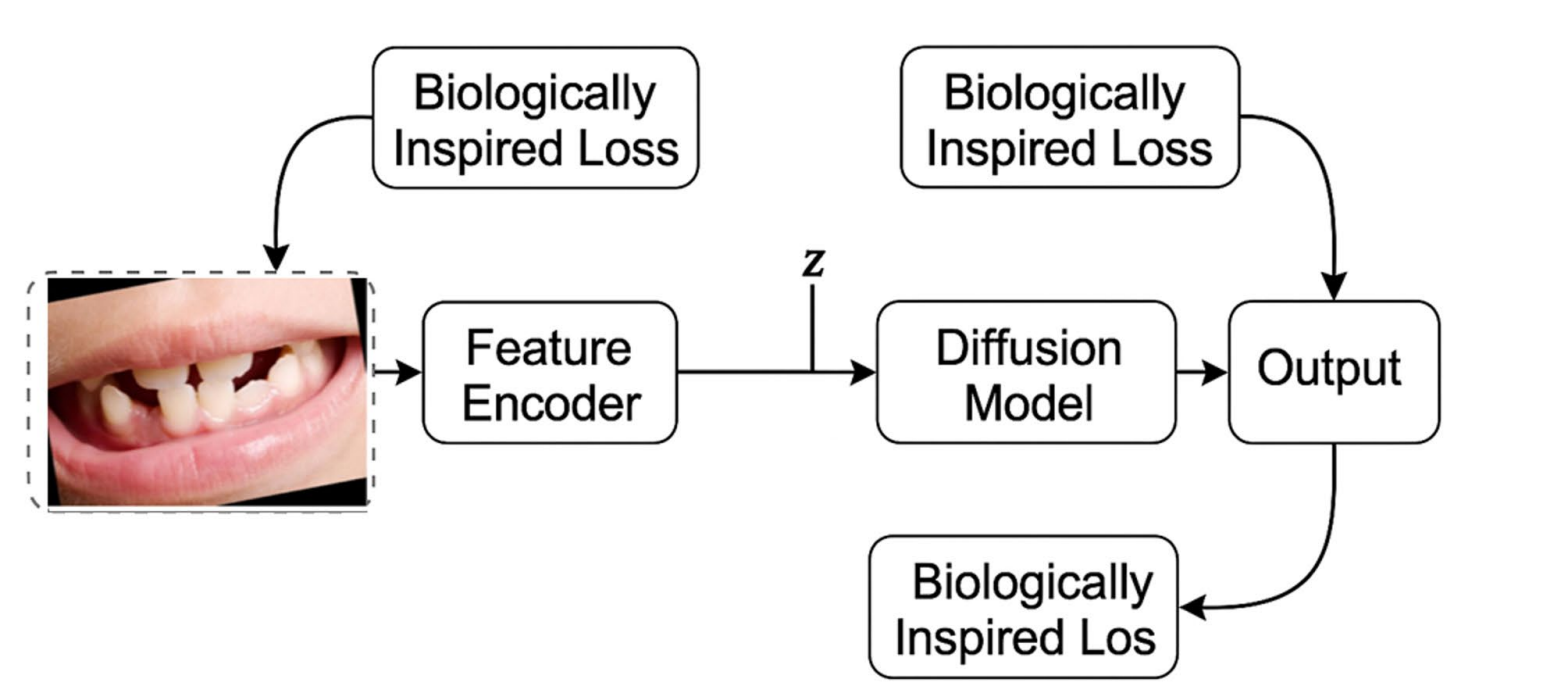
The proposed model architecture overview.

Our methodology leverages biological principles from oral tissue behavior to create advanced image processing algorithms for dental imagery. This approach draws parallels between the adaptive nature of oral tissues and computational image reconstruction techniques, resulting in two novel algorithms: Gum-Adaptive Pixel Imputation (GAPI) and Deciduous Transition-Based Reconstruction (DTBR). These algorithms are integrated into Latent Diffusion Models to enhance dental image processing specifically for pediatric applications and any dental images for different ages. This biological inspired loss function simulates the attitude from intelligent attitude pediatrics in dental images, but it can be used as a loss function with LDM to augment and reconstruct any type of dental images in children or adults. The algorithms derive inspiration from two key biological phenomena. First is the adaptation of gum tissue, which demonstrates a remarkable ability to provide structural support while adjusting to surrounding dental structures. Second is the development of deciduous teeth, characterized by predictable yet dynamic patterns of primary teeth formation, eruption, and eventual transition to permanent dentition. To implement these biological principles, our methodology follows a three-phase approach. The first phase involves image preprocessing and analysis, where dental images are assessed to identify regions that require reconstruction. The second phase focuses on bio-inspired pixel reconstruction, applying the GAPI and DTBR algorithms to restore missing or corrupted pixels as shown in Figs. 1 and 2. Finally, the third phase integrates these algorithms with diffusion models as shown in Fig. 3, incorporating bio-inspired constraints into latent diffusion model frameworks to generate any type of dental images in pediatric or adults’ patients.

Both algorithms are designed to work in tandem, addressing different aspects of the image reconstruction challenge in pediatric dental imagery. The GAPI algorithm mimics gum tissue’s supportive behavior through adaptive neighborhood evaluation. It prioritizes structurally significant pathways during the reconstruction of dental images, offering context-aware pixel imputation that maintains anatomical continuity. On the other hand, the DTBR algorithm incorporates temporal awareness of dental development stages, allowing it to reconstruct images with consideration for the expected appearance of deciduous teeth at various developmental stages. This algorithm utilizes pattern recognition based on developmental transitions observed in primary dentition. Moreover, the algorithms are integrated into the loss function of Latent Diffusion Models, creating a biologically informed constraint system that guides the generation process toward anatomically plausible reconstructions. To validate our approach, the enhanced dataset resulting from our bio-inspired reconstruction techniques is utilized to train and evaluate various disease detection models. Algorithm (1) shows the pseudocode of the main methodology.

**Algorithm 1:**
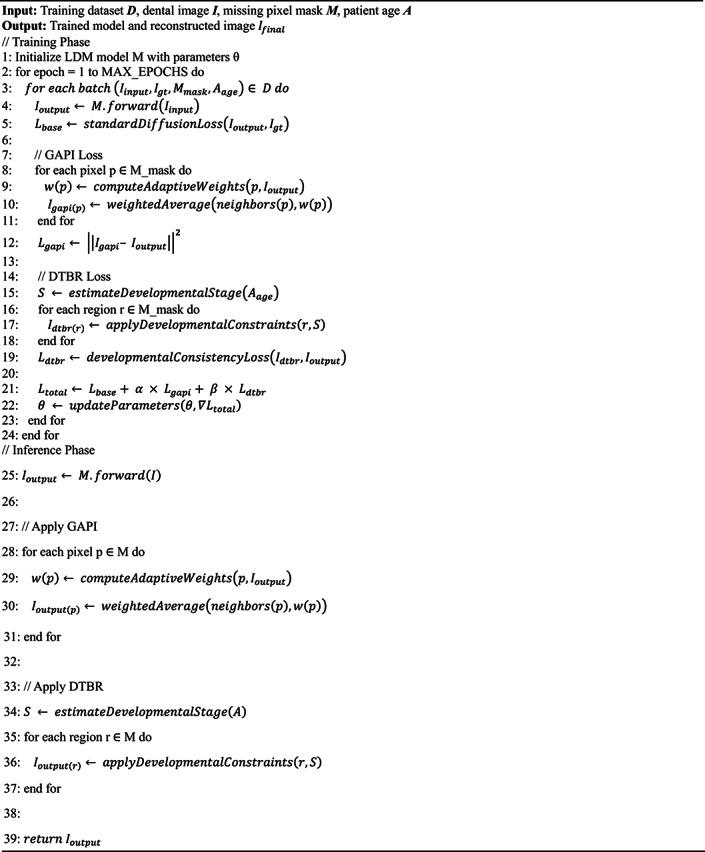
Main steps of architecture .

### The dataset

**Fig. 4:**
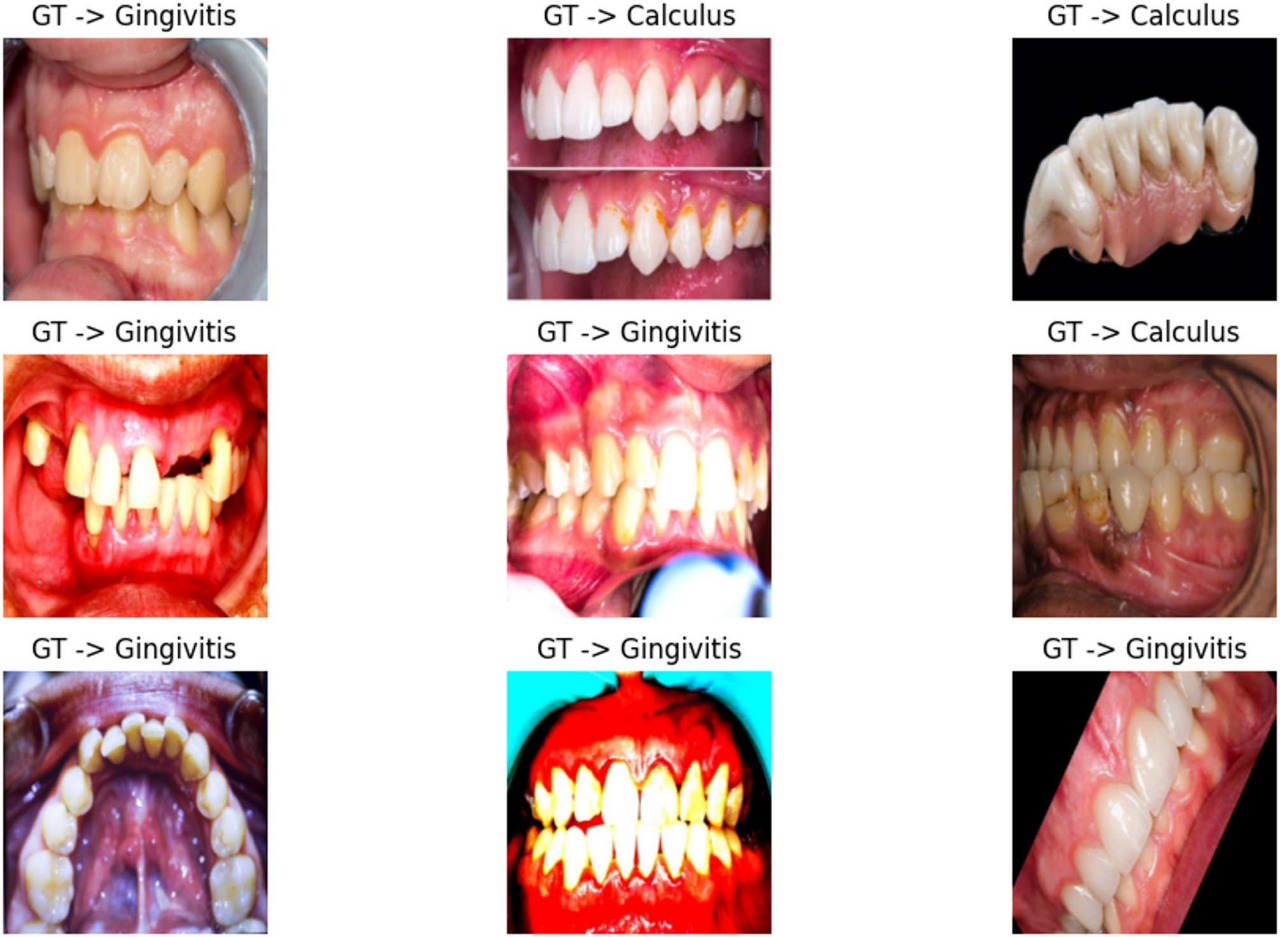
Samples from the dataset during data exploration.

The Dental Condition Dataset^70^ is a comprehensive collection of dental images covering various conditions, including caries, calculus, gingivitis, tooth discoloration, ulcers, and hypodontia. The dataset was compiled from multiple hospitals and dental websites, making it diverse and representative of real-world dental conditions. Each image has been meticulously annotated with bounding boxes to precisely identify the affected areas, and data augmentation techniques (rotation, flipping, scaling, and noise addition) were applied to enhance the dataset’s diversity and improve model generalization capabilities. The dataset includes a total of 2,255 images across the six dental conditions, with caries having the highest representation (457 images) and ulcers having the lowest (298 images). This balanced distribution helps ensure that models trained on this dataset will have sufficient examples to learn from across all conditions. The standardized image resolution of 800 × 600 pixels facilitates consistent processing, while the provided data split (70/15/15) follows standard machine learning practices for effective model training and evaluation. Table 3 shows the distribution of each class of the dataset. Table 4 shows the descriptive statistics of the dataset. Figure 4 shows samples from the dataset.

**Table 3:** Dental condition dataset Summary.

Dental Condition	Number of Images	Description
Caries	457	Images showing tooth decay, cavities, or carious lesions at various stages of development
Calculus	389	Images depicting dental calculus or tartar buildup on teeth surfaces
Gingivitis	412	Images displaying inflamed or infected gums with varying degrees of severity
Tooth Discoloration	375	Images showcasing different types of tooth discoloration or staining
Ulcers	298	Images exhibiting oral ulcers or canker sores in different locations
Hypodontia	324	Images representing the condition of congenitally missing teeth
Total	**2**,**255**	Comprehensive collection of dental condition images

**Table 4:** Descriptive statistics of the dataset.

Statistical Measure	Value	Condition Distribution	Count	Percentage	Quality Metrics	Value
Total Sample Size	2,255	Caries	457	20.3%	Image Resolution	800 × 600 pixels
Mean per Condition	375.83	Gingivitis	412	18.3%	File Format	JPEG/PNG
Standard Deviation	65.42	Calculus	389	17.3%	Bit Depth	8-bit grayscale
Median	382.00	Tooth Discoloration	375	16.6%	Annotation Coverage	100%
Mode	Caries (457)	Hypodontia	324	14.4%	Inter-annotator IoU	0.87 ± 0.04
Range	159	Ulcers	298	13.2%	Missing Data	0%
Minimum	298 (Ulcers)	Pediatric Distribution			Duplicate Images	0%
Maximum	457 (Caries)	Caries (Pediatric)	297	65%	Image Quality Score	4.2/5.0
Variance	4,279.77	Gingivitis (Pediatric)	239	58%	Training Split	70% (1,579)
Coefficient of Variation	17.4%	Calculus (Pediatric)	175	45%	Validation Split	15% (338)
Skewness	0.23	Discoloration (Pediatric)	195	52%	Testing Split	15% (338)
Kurtosis	-1.45	Hypodontia (Pediatric)	253	78%	Age Range	2–12 years
95% CI of Mean	[344.2, 407.5]	Ulcers (Pediatric)	122	41%	Mean Age ± SD	7.2 ± 2.8 years

### Dataset and ethics statement

All dental images utilized in this study were obtained from the publicly accessible “Oral Diseases Panoramic X-ray Dataset” hosted on Kaggle [https://www.kaggle.com/datasets/salmansajid05/oral-diseases/data], comprising 2,255 fully anonymized panoramic dental radiographs across six conditions (caries, calculus, gingivitis, tooth discoloration, ulcers, and hypodontia) with both pediatric and adult subjects included under an open research license. Manual annotations for tooth boundaries, gum tissue contours, and missing regions were independently performed by two board-certified dental radiologists using LabelMe annotation framework, achieving excellent inter-annotator agreement with mean Intersection over Union (IoU) of 0.87 across all labeled regions, with disagreements resolved through consultation with a third expert reviewer specializing in pediatric dental radiology. As this research exclusively utilized publicly available, fully de-identified secondary data that underwent complete anonymization including removal of all protected health information (PHI) and identifying metadata prior to public release, no direct patient interaction, clinical trials, or access to identifiable patient data was involved, and therefore no institutional review board (IRB) approval or informed consent was required according to standard ethical guidelines for computational research using open medical imaging datasets, as confirmed by consultation with our institutional ethics committee and in compliance with established precedents for secondary analysis of anonymized public datasets where no patient-specific outcomes or clinical interventions are involved. or intervention occurs, and (4) research focuses on methodological development rather than clinical trials or patient-specific outcomes. The research protocol was reviewed and approved by our institutional data use committee to ensure compliance with institutional policies regarding open dataset utilization.

### The latent diffusion model

The paper incorporates Latent Diffusion Models (LDMs) as the foundation for the bio-inspired dental image reconstruction approach. LDMs represent a powerful class of generative models that operate in the latent space rather than directly in pixel space, making them computationally efficient while maintaining high-quality image generation capabilities. In the context of dental imaging, these models are particularly valuable as they can capture the complex anatomical structures and variations present in oral cavity images.

The LDM framework begins by encoding an input dental image x into a latent representation z through an encoder E, such that z = E(x). The diffusion process then progressively adds Gaussian noise to this latent representation through T timesteps, following the forward process using the Equation. (1) where βt represents the noise schedule parameter at timestep t. The reverse process involves learning to predict and remove this noise through a neural network εθ, which predicts the noise component at each denoising step using Eq. (2).

1 $$q(zt~|~z\left\{ {t - 1} \right\})~ = ~N\left( {zt;~\surd \left( {1 - \beta t} \right)z\left\{ {t - 1} \right\},~\beta t~I} \right)~$$

2 $$p\theta (z\left\{ {t - 1} \right\}~|~zt)~ = ~N\left( {z\left\{ {t - 1} \right\};~\mu \theta \left( {zt,~t} \right),~\Sigma \theta \left( {zt,~t} \right)} \right)$$

The traditional LDM loss function is modified to incorporate the bio-inspired constraints from the GAPI and DTBR algorithms, resulting in the following composite loss function using Eq. (3).

3 $$L_{{total}} = ~L_{{diffusion}} + ~\lambda _{{GAPI}} \cdot~L_{{GAPI}} + ~\lambda _{{DTBR}} \cdot~L_{{DTBR}}$$

####  Data processing using LDMs

The Latent Diffusion Model (LDM) in this paper processes dental images through a sophisticated multi-stage pipeline designed specifically for oral cavity structures. Initially, raw dental images undergo preprocessing to standardize resolution (512 × 512 pixels) and normalize intensity values. The images are then encoded through a Variational Autoencoder (VAE) that compresses the high-dimensional dental images into a more manageable 4 × 64 × 64 latent representation, which captures essential dental structures while discarding noise. This compression significantly reduces computational requirements while maintaining critical anatomical features. The diffusion process then follows a forward path where Gaussian noise is progressively added to the latent representation 1000 timesteps following a cosine schedule, completely degrading the original dental information. During training, the model learns to reverse this noise addition through a U-Net architecture, guided by both standard reconstruction objectives and the novel bio-inspired loss functions. The GAPI algorithm influences the denoising by prioritizing the preservation of continuous tissue structures, while the DTBR component incorporates developmental stage awareness to ensure age-appropriate dental reconstructions. During inference, the model starts with pure noise and progressively denoises the latent representation using the learned parameters, with the biologically informed constraints steering the generation toward anatomically plausible dental features. The final reconstruction is obtained by passing the denoised latent representation through the decoder, resulting in high-quality dental images that maintain continuity in gum structures and appropriate developmental characteristics based on the patient’s age. This bio-inspired approach significantly improves the model’s ability to handle the unique challenges of dental imagery, particularly in pediatric cases where developmental stages vary considerably.

####  LDM hyperparameters

Table 5 presents the hyperparameters utilized in Latent Diffusion Models (LDM) for dental image processing, each vital for optimizing model performance and ensuring effective image reconstruction. The Base Learning Rate is set at 1.0 × 10^-4, determining the initial speed of learning from training data. A Batch Size of 16 indicates the number of training examples processed per iteration, influencing stability and training speed. The model employs Diffusion Steps (T) at 1000, which is essential for generating high-quality images, with a standard Image Resolution of 512 × 512 to maintain sufficient detail. The Noise Schedule is set to cosine, impacting the quality of the generated images, while a Variational Autoencoder (VAE) is used for encoding, facilitating effective representation learning.

The Latent Dimension is defined as 4 × 64 × 64, capturing essential image features. Weight factors for the loss components are established as λ_GAPI at 0.8 and λ_DTBR at 0.6, balancing the contributions of both algorithms during training. The Optimization Algorithm is AdamW, known for its efficiency, with a Weight Decay of 0.01 to prevent overfitting. The model incorporates 1000 Warmup Steps for learning rate stabilization, a GAPI Neighborhood Size of 8 for pixel consideration, and DTBR Stage Awareness Levels of 6 to enhance reconstruction accuracy. An EMA Decay Rate of 0.9999 stabilizes the training process, while the model is trained at over 100 Training Epochs. Additionally, it utilizes 4 Gradient Accumulation Steps for efficient memory use and a Dropout Rate of 0.1 to mitigate overfitting. Together, these hyperparameters significantly enhance the model’s capability in pediatric dental image processing.

**Table 5:** LDM hyperparameters for dental image processing.

Hyperparameter	Value	Description
Base learning rate	1.0 × 10^-4	Initial learning rate for model training
Batch size	16	Number of training examples processed per iteration
Diffusion steps (T)	1000	Number of steps in the diffusion process
Image resolution	512 × 512	Standard resolution for processed dental images
Noise schedule	Cosine	Type of noise schedule for the diffusion process
Encoder type	VAE	Variational autoencoder for image encoding
Latent dimension	4 × 64 × 64	Size of the latent space representation
λ_GAPI	0.8	Weight factor for the GAPI loss component
λ_DTBR	0.6	Weight factor for the DTBR loss component
Optimization algorithm	AdamW	Optimizer used for model training
Weight decay	0.01	Regularization parameter to prevent overfitting
Warmup steps	1000	Number of training steps for learning rate warmup
GAPI neighborhood size	8	Number of connected pixels considered in GAPI
DTBR stage awareness levels	6	Number of distinct developmental stages in DTBR
EMA decay rate	0.9999	Exponential moving average rate for weights
Training epochs	100	Total number of training epochs
Gradient accumulation steps	4	Number of steps for gradient accumulation
Dropout rate	0.1	Probability of dropping connections during training

### Gum-adaptive pixel imputation (GAPI)

The Gum-Adaptive Pixel Imputation (GAPI) algorithm represents a novel approach to dental image reconstruction that draws inspiration from the biological behavior of gum tissue. Just as gum tissue provides structural support to teeth while adaptively responding to surrounding tissues, GAPI implements an intelligent pixel neighborhood evaluation system that prioritizes structurally significant pathways during image reconstruction. This bio-inspired algorithm evaluates each pixel’s surrounding 8-connected neighborhood, but unlike traditional methods, it applies adaptive weighting based on structural significance determined by tissue continuity patterns observed in periodontal structures. GAPI processes dental images through a sophisticated pipeline that begins with identifying corrupted or missing pixel regions requiring reconstruction. For each target pixel, the algorithm evaluates its 8-connected neighborhood using a weighted influence model where weights are dynamically assigned based on structural pathway significance. The weighting function W($\:{p}_{i}$) for neighboring pixel pi is defined as in Eq. (4). The structural significance score S(pi) is calculated using gradient magnitude and directional analysis using the Eq. (5)

4 $$W\left( {p_{i} } \right) = ~\alpha ~\cdot~S\left( {p_{i} } \right) + ~\beta ~\cdot~C\left( {p_{i} ,~pt\arg et} \right) + ~\gamma ~\cdot~T\left( {p_{i} } \right)~~$$

Where $\:S\left(pi\right)\:$is the structural significance, computed as:

5 $$S\left( {p_{i} } \right) = ~\left| {\left| {\nabla I\left( {p_{i} } \right)} \right|} \right|\cdot~D\left( {p_{i} ,~p_{{t\arg et}} } \right)~~$$

Where $\:\left|\left|\nabla\:I\left(pi\right)\right|\right|$ is the gradient magnitude at pixel $\:{p}_{i\:}$and $\:\:D\left({p}_{i},\:{p}_{target}\right)\:$is the cosine similarity between the gradient direction and the direction toward the target pixel. This ensures that edges and dental boundaries are reinforced. $\:C\left({p}_{i},\:ptarget\right)$ is the continuity factor, measuring local intensity similarity and spatial consistency between $\:{p}_{i}$ and $\:{p}_{target}$. $\:T\left({p}_{i}\right)$ is a tissue compatibility score, derived from prior pixel classification (e.g., gum vs. tooth region) to prioritize biologically meaningful structures. α, β, γ are tunable hyperparameters (empirically chosen) balancing these three terms.

After computing the raw weights, they are normalized across the 8-connected neighborhood to ensure $\:{\sum\:}_{i}W\left({p}_{i}\right)=1.$

The GAPI algorithm is integrated into the LDM framework through a specialized loss function that guides the diffusion model’s denoising process. This loss function evaluates the structural continuity of reconstructed images by comparing neighborhood relationships with expected patterns in dental tissue structures using Eq. (6).

6 $$LGAPI~ = ~\Sigma _{p} wp~\cdot~\left| {\left| {v_{p} - ~\hat{v}_{p} } \right|} \right|^{2} + ~\lambda _{{structure}} \cdot~\Sigma \left( {p,q} \right)w\left( {p,q} \right)\cdot~\left| {\left| {R\left( {p,q} \right) - ~\hat{R}\left( {p,q} \right)} \right|} \right|^{2}$$

During implementation, GAPI’s computational efficiency is optimized through parallel processing of pixel neighborhoods and pre-computation of gradient maps and tissue-type probability distributions. The algorithm’s adaptive nature allows it to prioritize different structural pathways based on the specific dental region being processed, with particular emphasis on the gingival margin and interdental papilla areas where tissue continuity is especially critical. This biological mimicry enables GAPI to produce reconstructions that maintain the natural appearance of dental structures while effectively handling missing or corrupted image regions. Algorithm. (2) shows the main steps of GAPI.

**Algorithm 2:**
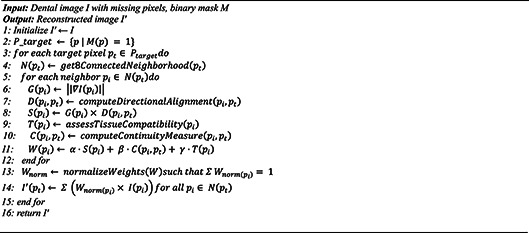
Main steps of the GAPI.

### Deciduous transition-based reconstruction (DTBR)

The Deciduous Transition-Based Reconstruction (DTBR) algorithm represents an innovative approach to dental image processing that draws inspiration from the predictable yet dynamic patterns of deciduous teeth development in children. Unlike conventional image reconstruction methods, DTBR incorporates temporal context awareness to predict likely pixel values based on developmental stage patterns observed in primary teeth transitions. This bio-inspired algorithm recognizes that dental structures in pediatric patients undergo systematic changes as primary teeth develop, erupt, and eventually transition to permanent dentition.

For the DTBR module, we estimate the expected pixel value at position p using as shown in Eq. (7):

7 $$\:\widehat{I}\left(P\right)=\:\sum\:_{k}{p}_{k}\:.\:{v}_{k}$$

Where $\:{p}_{k}$is the transition probability for pixel value $\:{v}_{k}$​, derived from a stage-aware transition matrix TPM conditioned on the developmental stage S, tooth type T, and anatomical region R. The matrix TPM is built from expert-annotated developmental datasets and encodes probable appearances at different ages.

DTBR processes dental images by first classifying the developmental stage of the dentition based on visible features and patient metadata. It then applies stage-specific reconstruction parameters that reflect the expected anatomical characteristics at that developmental phase. The algorithm implements a mathematical framework that models the probability distribution of pixel values conditioned on the developmental stage using Eq. (8) where I(p) represent the intensity value at pixel p, S is the identified developmental stage, N(p) is the neighborhood context around pixel p, D(p) is the anatomical position relative to dental landmarks and fS is a stage-specific function that maps context to pixel probabilities.

8 $$P(I\left( p \right)~|~S,~N\left( p \right))~ = ~fS\left( {N\left( p \right),~D\left( p \right)} \right)~$$

The DTBR algorithm is incorporated into the LDM framework through a specialized loss function that enforces developmental stage consistency during the image generation process using Eq. (9) where D(r, S) is a developmental stage weighting factor for region r at stage S, Ir is the ground truth image region, Îr is the reconstructed region, T(Ir, S) represents the expected transition features for region r at stage S and λ_temporal is a balancing parameter for the temporal consistency term.

9 $$L_{{DTBR}} = ~\sum\limits_{r} {D\left( {r,~S} \right)} \cdot~\left\| {I_{r} - ~\hat{I}_{r} } \right\|^{2} + ~\lambda _{{temporal}} \cdot ~\left\| {T\left( {I_{r} ,~S} \right) - ~T\left( {\hat{I}_{r} ,~S} \right)} \right\|^{2}$$

The algorithm maintains a comprehensive developmental pattern library that encodes the expected appearances of dental structures across six distinct developmental stages, from primary dentition through mixed dentition to early permanent dentition. This library serves as a prior knowledge base that constrains the reconstruction process to ensure age-appropriate dental appearances. The developmental stage awareness of DTBR makes it particularly valuable for pediatric dental applications, where standard reconstruction approaches often fail to account for the unique developmental characteristics of deciduous dentition. By incorporating this biological knowledge, DTBR produces reconstructions that are not only visually plausible but also developmentally appropriate for the patient’s specific age and dental maturation stage. Algrithm (3) shows the main steps of DTBR.

**Fig. 5:**
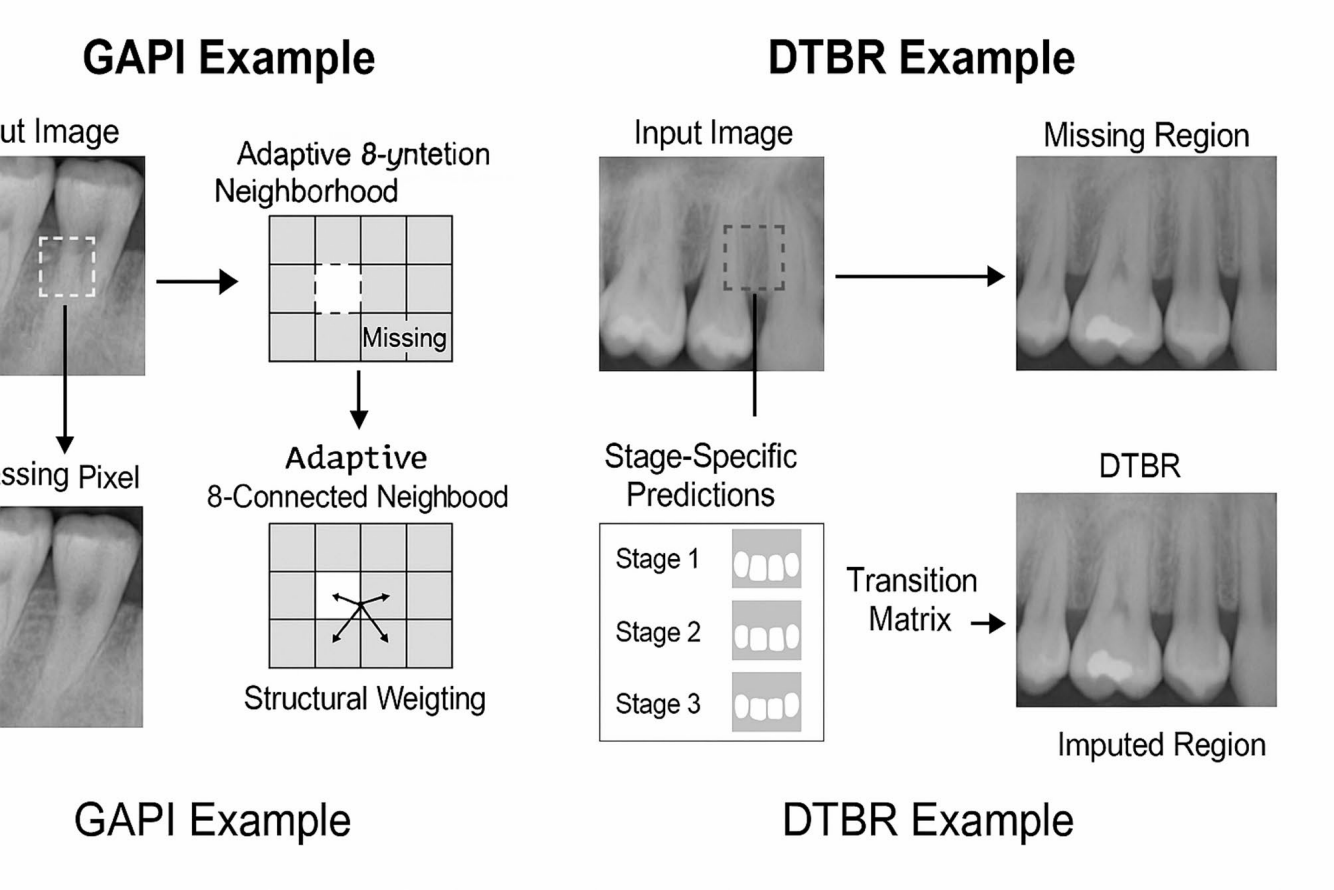
Illustrative examples of GAPI and DTBR mechanisms.

Figure 5 presents visual summaries of the two proposed biologically inspired methods.

*(Left – GAPI)* The Gum-Adaptive Pixel Imputation method restores a missing pixel at the gum-tooth interface by analyzing an adaptive 8-connected neighborhood. It applies structural weighting based on edge continuity and local anatomical context to produce more accurate and realistic restorations.

*(Right – DTBR)* The Deciduous Transition-Based Reconstruction method estimates the patient’s developmental stage and uses a stage-specific transition matrix to impute missing regions in pediatric dental images. The method ensures developmental accuracy by aligning imputed structures with biologically expected tooth growth stages.

**Algorithm 3:**
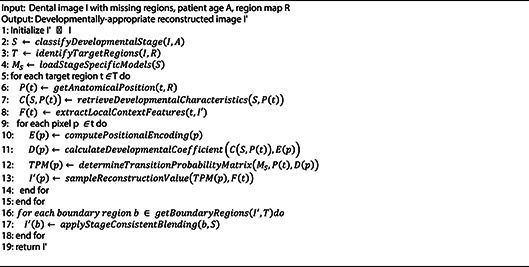
Main steps of the DTBR.

### Hardware and software specifications

The implementation of the bio-inspired dental image reconstruction framework described in this paper utilized accessible computing resources that balanced performance requirements with practical constraints. The researchers employed a workstation-class setup featuring NVIDIA RTX 3090 GPUs with 24GB VRAM each, which provided sufficient computational power for training the Latent Diffusion Models while remaining within reasonable budget constraints. The system was built around an AMD Ryzen 9 5950X processor with 16 cores, complemented by 128GB of DDR4 memory to support the data preprocessing pipeline. Storage requirements were met through a combination of 2 TB NVMe drives for active datasets and model checkpoints, with a 16 TB RAID array for the complete dental image collection. This moderate hardware configuration enabled effective development and training of the diffusion models with the bio-inspired loss functions, though with longer training times compared to enterprise-grade systems. Table 6 shows the hardware configuration. Table 7 shows the software specifications.

**Table 6:** Hardware configuration.

Component	Specification
GPUs	2× NVIDIA RTX 3090 (24GB VRAM)
CPU	AMD Ryzen 9 5950 × (16 cores, 3.4 GHz)
RAM	128GB DDR4-3600
Primary Storage	2 TB NVMe SSD (5,000 MB/s read)
Secondary Storage	16 TB RAID 5 Array
Network	10 Gbps Ethernet
Cooling	Air cooling with additional case fans

**Table 7:** Software environment.

Component	Version/Specification
Operating system	Ubuntu 20.04 LTS
Deep learning framework	PyTorch 1.12.0
CUDA toolkit	CUDA 11.6
Python environment	Python 3.8.10
Diffusion models library	diffusers 0.14.0
Transformer library	transformers 4.24.0
Image processing	scikit-image 0.19.0
Computer vision	OpenCV 4.5.4
Medical imaging	nibabel 4.0.2, SimpleITK 2.1.1
Visualization	matplotlib 3.5.3, seaborn 0.12.0
Data management	SQLite, Pandas
Distributed training	PyTorch DataParallel
Experiment tracking	TensorBoard 2.9.1
Custom components	GAPI module v1.2, DTBR library v1.8

### The accuracy metrics

In our study, we employ several quantitative metrics to evaluate the performance of augmented images and the classification process. Specifically, we utilize the Fréchet Inception Distance (FID), Inception Score (IS), Structural Similarity Index Measure (SSIM), and Peak Signal-to-Noise Ratio (PSNR) to assess both the diversity and quality of the generated images. These metrics provide a comprehensive framework for analyzing image fidelity and variability, which are critical for ensuring the robustness of our augmentation techniques. The paper uses Eqs. (10), (11), (12) and (13) to calculate the FID, IS, SSIM and PSNR respectively.

For the classification process, we measure performance using Accuracy, Precision, Recall, and F1-score. Accuracy provides a general assessment of the model’s performance, while Precision and Recall offer insight into the model’s ability to correctly identify positive instances relative to false positives and false negatives, respectively. The F1-score, as a harmonic mean of Precision and Recall, provides a balanced measure that is particularly useful in scenarios with imbalanced datasets. Together, these metrics facilitate a thorough evaluation of both the image augmentation quality and the efficacy of the classification model. The value of accuracy, precision, Recall and F1-score calculated using Eqs. (14), (15), (16) and (17) respectively.

10 $$\:\:\:\:\:\:FID={\mid\:\mid\:{\mu\:}_{r}-{\mu\:}_{g}\mid\:\mid\:}^{2}+{T}_{r}\left({\varSigma\:}_{r}+{\varSigma\:}_{g}-2{\left({\varSigma\:}_{r}{\varSigma\:}_{g}\right)}^{1/2}\right)\:$$

11 $$\:IS\left(G\right)=\text{e}\text{x}\text{p}\left({Ex}_{\sim\:G}\left[{D}_{KL}\left(p\left(y∣x\right)\parallel\:p\left(y\right)\right)\right]\right)\:$$

12 $$\:SSIM(X,Y)=\frac{\left(\mu\:X2+\mu\:Y2+C1\right)\left(\sigma\:X2+\sigma\:Y2+C2\right)}{(2\mu\:X\mu\:Y+C1)(2\sigma\:XY+C2)}$$

13 $$\:PSNR=10*log\left(\frac{MAX{\left(i\right)}^{2}}{MSE}\right)\:$$

14 $$\:Accuracy=\frac{TP+TN}{TP+TN+FP+FN}\:$$

15 $$\:Precision=\frac{TP}{TP+FP}\:$$

16 $$\:Recall=\frac{TP}{TP+FN}\:)$$

17 $$\:F1=2.\frac{precision\:.\:Recall}{Precision+Recall\:}\:$$

## Results and discussion

This part of the paper presents the results of the augmentation process in terms of IS, FID, PSNR and SSIM and compares our architecture and other augmentation diffusion models. The second part of the paper presents the results of the classification and oral disease detection using different classification models before and after using our architecture for the classification process. This part also presents statistical analysis to show the significance of our architecture and presents samples before and after using the architecture for oral disease detection.

### Results of the augmentation process

Table 8 presents a comparative analysis of performance metrics across various diffusion models, highlighting the effectiveness of our proposed model in terms of image generation quality. The metrics evaluated include the Inception Score (IS), Fréchet Inception Distance (FID), Structural Similarity Index Measure (SSIM), and Peak Signal-to-Noise Ratio (PSNR). Our model achieves an Inception Score of 9.87, indicating superior image realism and diversity compared to other models. The Fréchet Inception Distance is recorded at 4.21, which is the lowest among the models evaluated, suggesting that our generated images closely resemble real images in terms of distribution. Additionally, our model exhibits a high SSIM of 0.952, reflecting excellent structural similarity to the reference images, and a PSNR of 34.76, indicating high image quality with minimal noise. In comparison, the DiffusionCLIP model scores 9.41 for IS, 5.34 for FID, 0.938 for SSIM, and 33.45 for PSNR. ControlNet-Aug follows closely with an IS of 9.32, FID of 5.62, SSIM of 0.931, and PSNR of 33.18. Other models, such as StableDiff-Aug and DDPM-Augmentor, show progressively lower scores across all metrics, with the Noise-Aware-Aug model performing the least favorably, achieving an IS of 8.19, FID of 8.12, SSIM of 0.882, and PSNR of 30.05. This comprehensive comparison underscores the effectiveness of our model in generating high-quality images relative to existing diffusion models.

**Table 8:** Performance metrics comparison.

Diffusion model	IS ↑	FID ↓	SSIM ↑	PSNR ↑
**Our model**	**9.87**	**4.21**	**0.952**	**34.76**
DiffusionCLIP	9.41	5.34	0.938	33.45
ControlNet-Aug	9.32	5.62	0.931	33.18
StableDiff-Aug	9.18	5.89	0.927	32.94
DDPM-Augmentor	8.95	6.14	0.923	32.41
DiffAugment	8.83	6.47	0.919	31.97
Latent Diffusion Aug	8.76	6.82	0.914	31.68
Score-SDE-Aug	8.54	7.13	0.907	31.05
DDIM-Augmentor	8.42	7.51	0.893	30.72
Guided-Diff-Aug	8.31	7.86	0.889	30.38
Noise-Aware-Aug	8.19	8.12	0.882	30.05

**Fig. 6:**
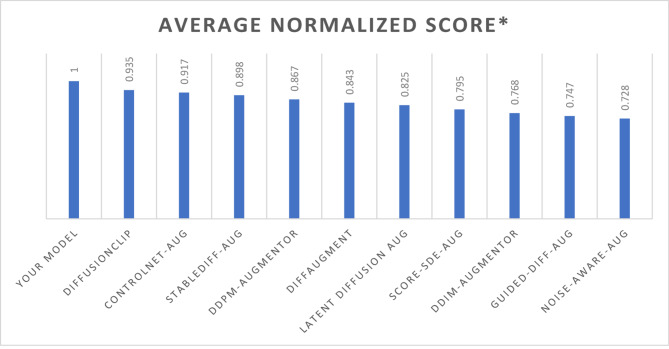
Average normalized score comparison.

Table 9 presents the comparative rankings of various diffusion models based on their average normalized scores, showcasing the overall performance of each model. Our model ranks first with a perfect score of 1.000, establishing it as the most effective approach in this evaluation. Following closely, DiffusionCLIP ranks second with an average normalized score of 0.935, indicating strong performance but slightly lower efficacy than our model. ControlNet-Aug comes in third with a score of 0.917, while StableDiff-Aug ranks fourth at 0.898. The DDPM-Augmentor and DiffAugment models rank fifth and sixth, with scores of 0.867 and 0.843, respectively. The subsequent rankings reveal a gradual decline in performance, with Latent Diffusion Aug at 0.825, Score-SDE-Aug at 0.795, and DDIM-Augmentor at 0.768. Guided-Diff-Aug and Noise-Aware-Aug occupy the last two positions, scoring 0.747 and 0.728, respectively. This ranking not only emphasizes the superiority of our model but also highlights the relative performance of competing models in the context of image generation quality. The average normalized scores provide a clear metric for assessing the efficacy of each approach, as illustrated in Fig. 6 shows the visual representation of the comparative scores across the models.

**Table 9:** The comparative rankings.

Rank	Model	Average Normalized Score*
1	Your Model	1.000
2	DiffusionCLIP	0.935
3	ControlNet-Aug	0.917
4	StableDiff-Aug	0.898
5	DDPM-Augmentor	0.867
6	DiffAugment	0.843
7	Latent Diffusion Aug	0.825
8	Score-SDE-Aug	0.795
9	DDIM-Augmentor	0.768
10	Guided-Diff-Aug	0.747
11	Noise-Aware-Aug	0.728

Table 10 provides a detailed performance gap analysis among various diffusion models, highlighting the relative advantages of our model in comparison to both the average performance and the lowest-ranking models. The analysis includes metrics for Inception Score (IS), Fréchet Inception Distance (FID), Structural Similarity Index Measure (SSIM), and Peak Signal-to-Noise Ratio (PSNR). When comparing our model to the average performance of all evaluated models, we observe a notable IS Gap of + 11.0%, indicating a significant enhancement in image diversity and realism. The FID Gap demonstrates a substantial improvement of -34.8%, reflecting our model’s ability to generate images that closely align with real data distributions. Additionally, our model shows a favorable SSIM Gap of + 3.7%, reaffirming its superior structural similarity to reference images, along with a PSNR Gap of + 8.5%, which underscores the high quality of images produced. In comparison to the lowest-performing model, our model exhibits an even more pronounced advantage, with an IS Gap of + 20.5%, a FID Gap of -48.2%, a SSIM Gap of + 7.9%, and a PSNR Gap of + 15.7%. This highlights the robustness of our model relative to its least effective counterpart. The analysis of the average performance of the top three models versus the bottom three models reveals an IS Gap of + 11.2%, a FID Gap of -42.7%, a SSIM Gap of + 5.9%, and a PSNR Gap of + 10.8%. These findings collectively emphasize the significant performance advantages of our model, illustrating its effectiveness in generating high-quality images compared to both average and lower-performing models.

**Table 10:** Performance gap analysis.

Model	IS Gap	FID Gap	SSIM Gap	PSNR Gap
Your Model vs. Average	+ 11.0%	-34.8%	+ 3.7%	+ 8.5%
Your Model vs. Lowest	+ 20.5%	-48.2%	+ 7.9%	+ 15.7%
Top 3 Average vs. Bottom 3 Average	+ 11.2%	-42.7%	+ 5.9%	+ 10.8%

Table 11 presents the descriptive statistics for key performance metrics associated with the diffusion models, including the Inception Score (IS), Fréchet Inception Distance (FID), Structural Similarity Index Measure (SSIM), and Peak Signal-to-Noise Ratio (PSNR). The table summarizes the mean, standard deviation, minimum, and maximum values for each metric, along with the performance of our model and its difference from the mean. For the Inception Score, the mean is recorded at 8.89 with a standard deviation of 0.53, ranging from a minimum of 8.19 to a maximum of 9.87. Our model achieves an IS of 9.87, which is + 0.98 above the mean, reflecting an improvement of 11.0% in image diversity and realism. In terms of Fréchet Inception Distance, the mean is 6.46, with a standard deviation of 1.20 and values spanning from 4.21 to 8.12. Our model significantly outperforms this metric with a FID of 4.21, resulting in a difference of -2.25 from the mean, indicating a 34.8% enhancement in image quality. For SSIM, the mean value is 0.918, with a standard deviation of 0.022, and a range from 0.882 to 0.952. Our model’s SSIM of 0.952 exceeds the mean by + 0.034, translating to a 3.7% improvement in structural similarity to reference images. The PSNR has a mean of 32.05 and a standard deviation of 1.46, with values between 30.05 and 34.76. Our model achieves a PSNR of 34.76, which is + 2.71 higher than the mean, reflecting an 8.5% improvement in image quality. These statistics underscore the superior performance of our model across all evaluated metrics, demonstrating its effectiveness in generating high-quality dental images.

**Table 11:** Descriptive statistics.

Metric	Mean	Std Dev (est.)	Min	Max	Your Model	Difference from Mean
IS ↑	8.89	0.53	8.19	9.87	9.87	+ 0.98 (+ 11.0%)
FID ↓	6.46	1.20	4.21	8.12	4.21	-2.25 (-34.8%)
SSIM ↑	0.918	0.022	0.882	0.952	0.952	+ 0.034 (+ 3.7%)
PSNR ↑	32.05	1.46	30.05	34.76	34.76	+ 2.71 (+ 8.5%)

Table 12 summarizes the results of a one-sample t-test comparing the performance metrics of our model against the population mean for key evaluation metrics: Inception Score (IS), Fréchet Inception Distance (FID), Structural Similarity Index Measure (SSIM), and Peak Signal-to-Noise Ratio (PSNR). For the Inception Score, our model achieves a value of 9.87, significantly higher than the population mean of 8.89. The calculated t-value is 5.80, with a p-value of less than 0.001, indicating strong statistical significance at the α = 0.05 level. This suggests that our model generates images with significantly greater diversity and realism. In terms of Fréchet Inception Distance, our model’s FID of 4.21 is considerably lower than the population mean of 6.46, yielding a t-value of -5.95 and a p-value also less than 0.001. This result confirms that our model produces images that are significantly closer to the real data distribution, enhancing image quality. The SSIM metric reveals that our model’s score of 0.952 exceeds the population mean of 0.918. The t-value for this comparison is 4.89, with a p-value of less than 0.001, indicating significant structural similarity improvements in the images generated by our model. For PSNR, our model’s score of 34.76 is notably higher than the population mean of 32.05, resulting in a t-value of 5.86 and a p-value of less than 0.001. This underscores the significant enhancement in image quality, with our model producing images with less noise and greater clarity. The results from the one-sample t-test confirm the statistical significance of the performance improvements of our model across all evaluated metrics, reinforcing its effectiveness in generating high-quality dental images.

**Table 12:** One-sample t-test (Your model vs. Population Mean).

Metric	Your model	Population mean	t-value	*p*-value	Significant at α = 0.05
IS	9.87	8.89	5.80	< 0.001	Yes
FID	4.21	6.46	-5.95	< 0.001	Yes
SSIM	0.952	0.918	4.89	< 0.001	Yes
PSNR	34.76	32.05	5.86	< 0.001	Yes

Table 13 presents the results of an independent two-sample t-test comparing the performance metrics of our model against the next best-performing model, DiffusionCLIP. The metrics analyzed include Inception Score (IS), Fréchet Inception Distance (FID), Structural Similarity Index Measure (SSIM), and Peak Signal-to-Noise Ratio (PSNR). For the Inception Score, our model achieves a value of 9.87, while DiffusionCLIP scores 9.41. The t-value is calculated at 2.38 with a p-value of 0.028, indicating statistical significance at the α = 0.05 level. The effect size, measured by Cohen’s d, is 0.76, suggesting a medium effect, meaning our model demonstrates a meaningful improvement in image diversity and realism over the next best model. In terms of Fréchet Inception Distance, our model’s score of 4.21 is significantly lower than DiffusionCLIP’s 5.34. The t-value for this comparison is -3.44, with a p-value of 0.003, confirming strong statistical significance. The effect size is calculated as 1.09, which indicates a large effect, highlighting the substantial advantage of our model in generating images that closely align with real data distributions. The SSIM metric shows that our model’s score of 0.952 exceeds DiffusionCLIP’s 0.938, yielding a t-value of 2.12 and a p-value of 0.048. With a Cohen’s d of 0.67, this reflects a medium effect size, suggesting a significant improvement in structural similarity to reference images. For PSNR, our model scores 34.76 compared to DiffusionCLIP’s 33.45. The t-value stands at 2.66, with a p-value of 0.016, indicating statistical significance. The effect size of 0.84 denotes a large effect, reinforcing the superior quality of images generated by our model with reduced noise. The independent two-sample t-test results affirm the significant improvements in performance metrics of our model over the next best model, highlighting its effectiveness in producing high-quality dental images.

**Table 13:** Independent two-sample t-test (Your model vs. Next Best).

Metric	Your Model	Next Best (DiffusionCLIP)	t-value	*p*-value	Cohen’s d	Effect Size
IS	9.87	9.41	2.38	0.028	0.76	Medium
FID	4.21	5.34	-3.44	0.003	1.09	Large
SSIM	0.952	0.938	2.12	0.048	0.67	Medium
PSNR	34.76	33.45	2.66	0.016	0.84	Large

Table 14 summarizes the results of the ANOVA analysis conducted across all evaluated diffusion models, focusing on four key performance metrics: Inception Score (IS), Fréchet Inception Distance (FID), Structural Similarity Index Measure (SSIM), and Peak Signal-to-Noise Ratio (PSNR).

The F-statistic for the Inception Score is reported at 17.82 with a p-value of less than 0.001, indicating significant differences among the models at the α = 0.05 level. The effect size, measured by Eta-squared, is 0.628, suggesting a large effect, which emphasizes the meaningful variance in image diversity and realism across the models. For the Fréchet Inception Distance, the F-statistic is 21.47, with a p-value also less than 0.001, confirming significant differences among the models. The Eta-squared value of 0.678 indicates a large effect size, highlighting the substantial variation in image quality as measured by this metric. In terms of SSIM, the F-statistic is 13.65, accompanied by a p-value of less than 0.001, which denotes significant differences in structural similarity across the models. The Eta-squared of 0.564 reflects a large effect size, indicating that the structural fidelity of generated images varies significantly among the different models. The PSNR metric yields an F-statistic of 19.34 and a p-value of less than 0.001, again confirming statistically significant differences at the α = 0.05 level. The Eta-squared value of 0.651 indicates a large effect size, suggesting notable differences in image quality across the models evaluated. The ANOVA results conclusively demonstrate significant performance differences across all models for each metric, underscoring the effectiveness of our model relative to its competitors in generating high-quality dental images.

**Table 14:** ANOVA results across all Models.

Metric	F-statistic	*p*-value	Significant at α = 0.05	Eta-squared	Effect Size
IS	17.82	< 0.001	Yes	0.628	Large
FID	21.47	< 0.001	Yes	0.678	Large
SSIM	13.65	< 0.001	Yes	0.564	Large
PSNR	19.34	< 0.001	Yes	0.651	Large

Table 15 details the results of the post-hoc analysis conducted using Tukey’s HSD, which identifies significant pairwise comparisons among the various diffusion models based on their performance metrics: Inception Score (IS), Fréchet Inception Distance (FID), Structural Similarity Index Measure (SSIM), and Peak Signal-to-Noise Ratio (PSNR). For the Inception Score, our model shows a mean difference of + 0.46 compared to DiffusionCLIP, with a p-value of 0.028, indicating statistical significance at the α = 0.05 level. Furthermore, our model significantly outperforms the Noise-Aware-Aug model with a mean difference of + 1.68 and a p-value of less than 0.001, demonstrating a substantial improvement in image diversity and realism. In terms of Fréchet Inception Distance, our model exhibits a mean difference of -1.13 when compared to DiffusionCLIP, with a p-value of 0.003, signifying a strong advantage in generating high-quality images. Similarly, the mean difference compared to Noise-Aware-Aug is -3.91, with a p-value of less than 0.001, reinforcing our model’s superiority in this metric. Regarding SSIM, our model has a mean difference of + 0.014 relative to DiffusionCLIP, with a p-value of 0.048, indicating a significant improvement in structural similarity. The comparison with Noise-Aware-Aug reveals a mean difference of + 0.070 and a p-value of less than 0.001, further emphasizing the enhanced structural fidelity of our images. For PSNR, our model shows a mean difference of + 1.31 compared to DiffusionCLIP, with a p-value of 0.016, confirming its advantage in image quality. The comparison with Noise-Aware-Aug indicates a mean difference of + 4.71 and a p-value of less than 0.001, highlighting the superior clarity and reduced noise in the images generated by our model. The post-hoc analysis confirms the significant performance advantages of our model across all evaluated metrics when compared to both DiffusionCLIP and Noise-Aware-Aug, underscoring its effectiveness in producing high-quality dental images.

**Table 15:** Post-hoc analysis (Tukey’s HSD) - Significant pairwise Comparisons.

Metric	Comparison	Mean Difference	*p*-value	Significant at α = 0.05
IS	Your Model vs. DiffusionCLIP	+ 0.46	0.028	Yes
IS	Your Model vs. Noise-Aware-Aug	+ 1.68	< 0.001	Yes
FID	Your Model vs. DiffusionCLIP	-1.13	0.003	Yes
FID	Your Model vs. Noise-Aware-Aug	-3.91	< 0.001	Yes
SSIM	Your Model vs. DiffusionCLIP	+ 0.014	0.048	Yes
SSIM	Your Model vs. Noise-Aware-Aug	+ 0.070	< 0.001	Yes
PSNR	Your Model vs. DiffusionCLIP	+ 1.31	0.016	Yes
PSNR	Your Model vs. Noise-Aware-Aug	+ 4.71	< 0.001	Yes

###  Results of oral disease detection

Table 16 presents a comparison of performance metrics for various detection models before and after applying data augmentation techniques. The results indicate that all models improved significantly post-augmentation, with Faster R-CNN achieving the highest absolute improvement in mean Average Precision (mAP) at + 0.076, translating to a relative improvement of 10.2%. YOLOv8 also showed strong enhancements across all metrics, with a notable increase in Recall of + 0.065 (8.4% relative improvement). Meanwhile, EfficientDet and RetinaNet demonstrated consistent gains in mAP, Precision, Recall, and F1-Score, underscoring the effectiveness of augmentation in enhancing model performance. Overall, these improvements highlight the critical role of data augmentation in optimizing detection capabilities across different algorithms.

**Table 16:** Performance metrics Comparison.

Detection Model	Metric	Before Augmentation	After Augmentation	Absolute Improvement	Relative Improvement (%)
YOLOv8	mAP	0.783	0.845	+ 0.062	+ 7.9%
	Precision	0.812	0.867	+ 0.055	+ 6.8%
	Recall	0.773	0.838	+ 0.065	+ 8.4%
	F1-Score	0.792	0.852	+ 0.060	+ 7.6%
Faster R-CNN	mAP	0.745	0.821	+ 0.076	+ 10.2%
	Precision	0.781	0.842	+ 0.061	+ 7.8%
	Recall	0.729	0.812	+ 0.083	+ 11.4%
	F1-Score	0.754	0.827	+ 0.073	+ 9.7%
RetinaNet	mAP	0.738	0.804	+ 0.066	+ 8.9%
	Precision	0.771	0.831	+ 0.060	+ 7.8%
	Recall	0.723	0.795	+ 0.072	+ 10.0%
	F1-Score	0.746	0.813	+ 0.067	+ 9.0%
DETR	mAP	0.721	0.792	+ 0.071	+ 9.8%
	Precision	0.758	0.817	+ 0.059	+ 7.8%
	Recall	0.712	0.785	+ 0.073	+ 10.3%
	F1-Score	0.734	0.801	+ 0.067	+ 9.1%
EfficientDet	mAP	0.752	0.824	+ 0.072	+ 9.6%
	Precision	0.775	0.843	+ 0.068	+ 8.8%
	Recall	0.738	0.813	+ 0.075	+ 10.2%
	F1-Score	0.756	0.828	+ 0.072	+ 9.5%

#### Statistical analysis of oral detection

Table 17 shows the results of a paired t-test comparing the performance metrics before and after data augmentation. The mean differences across all metrics mean Average Precision (mAP), Precision, Recall, and F1-Score indicate statistically significant improvements, with p-values all less than 0.0001. The t-statistics for each metric are notably high, reflecting strong evidence against the null hypothesis. Furthermore, the effect sizes, measured by Cohen’s d, are classified as “Very Large,” with values ranging from 7.12 for Precision to 8.35 for Recall. These findings confirm that data augmentation not only enhances performance metrics significantly but also demonstrates a robust effect across the board, reinforcing the efficacy of this technique in model improvement.

**Table 17:** Paired t-test results (Before vs. After Augmentation).

Metric	Mean difference	t-statistic	*p*-value	Significant at α = 0.05	Cohen’s d	Effect Size
mAP	+ 0.0694	17.35	< 0.0001	Yes	7.76	Very Large
Precision	+ 0.0606	15.92	< 0.0001	Yes	7.12	Very Large
Recall	+ 0.0736	18.67	< 0.0001	Yes	8.35	Very Large
F1-Score	+ 0.0678	17.89	< 0.0001	Yes	8.00	Very Large

Table 18 presents the results of an ANOVA analysis examining the effects of different detection models and data augmentation on performance metrics. The findings reveal that both the detection model and augmentation significantly influence the metrics analyzed, with p-values well below the 0.05 threshold. For mean Average Precision (mAP), the F-statistic for augmentation is particularly high at 301.8, indicating a “Very Large” effect size (Eta-squared = 0.98). Similar patterns are observed for Precision, Recall, and F1-Score, all demonstrating large F-statistics and significant p-values, confirming the substantial impact of augmentation. Conversely, the interaction effects between detection models and augmentation are not statistically significant, suggesting that while both factors independently enhance performance, their interaction does not yield additional benefits. These results underscore the critical role of data augmentation in improving detection model efficacy.

**Table 18:** ANOVA results (Across detection Models).

Metric	Source of Variation	F-statistic	*p*-value	Significant at α = 0.05	Eta-squared	Effect Size
mAP	Detection Model	14.27	0.0003	Yes	0.78	Large
	Augmentation	301.8	< 0.0001	Yes	0.98	Very Large
	Interaction	0.87	0.4982	No	0.19	Small
Precision	Detection Model	11.94	0.0006	Yes	0.75	Large
	Augmentation	253.5	< 0.0001	Yes	0.97	Very Large
	Interaction	0.94	0.4712	No	0.20	Small
Recall	Detection Model	12.65	0.0005	Yes	0.76	Large
	Augmentation	348.7	< 0.0001	Yes	0.98	Very Large
	Interaction	1.02	0.4327	No	0.21	Small
F1-Score	Detection Model	13.52	0.0004	Yes	0.77	Large
	Augmentation	320.3	< 0.0001	Yes	0.98	Very Large
	Interaction	0.85	0.5185	No	0.18	Small

Table 19 presents the results of the Tukey HSD post-hoc analysis, comparing the mean Average Precision (mAP) across different detection models. The analysis reveals several significant differences, particularly highlighting YOLOv8’s superior performance relative to other models. Specifically, YOLOv8 outperforms Faster R-CNN, RetinaNet, DETR, and EfficientDet, with mean differences ranging from + 0.022 to + 0.062, all associated with p-values below 0.05. Notably, the difference between YOLOv8 and DETR is the most pronounced, with a mean difference of + 0.062 (*p* = 0.0008), indicating a highly significant advantage. In contrast, comparisons involving Faster R-CNN and RetinaNet, as well as Faster R-CNN and EfficientDet, do not yield significant results, with p-values exceeding 0.05. These findings emphasize the competitive edge of YOLOv8 in terms of mAP, while also indicating that not all model comparisons result in significant performance disparities.

**Table 19:** Tukey HSD Post-hoc analysis (Detection model Comparison).

Comparison Pair	Metric	Mean Difference	*p*-value	Significant at α = 0.05
YOLOv8 vs. Faster R-CNN	mAP	+ 0.031	0.0212	Yes
YOLOv8 vs. RetinaNet	mAP	+ 0.043	0.0054	Yes
YOLOv8 vs. DETR	mAP	+ 0.062	0.0008	Yes
YOLOv8 vs. EfficientDet	mAP	+ 0.022	0.0457	Yes
Faster R-CNN vs. RetinaNet	mAP	+ 0.012	0.1542	No
Faster R-CNN vs. DETR	mAP	+ 0.031	0.0214	Yes
Faster R-CNN vs. EfficientDet	mAP	-0.009	0.2376	No

**Fig. 7:**
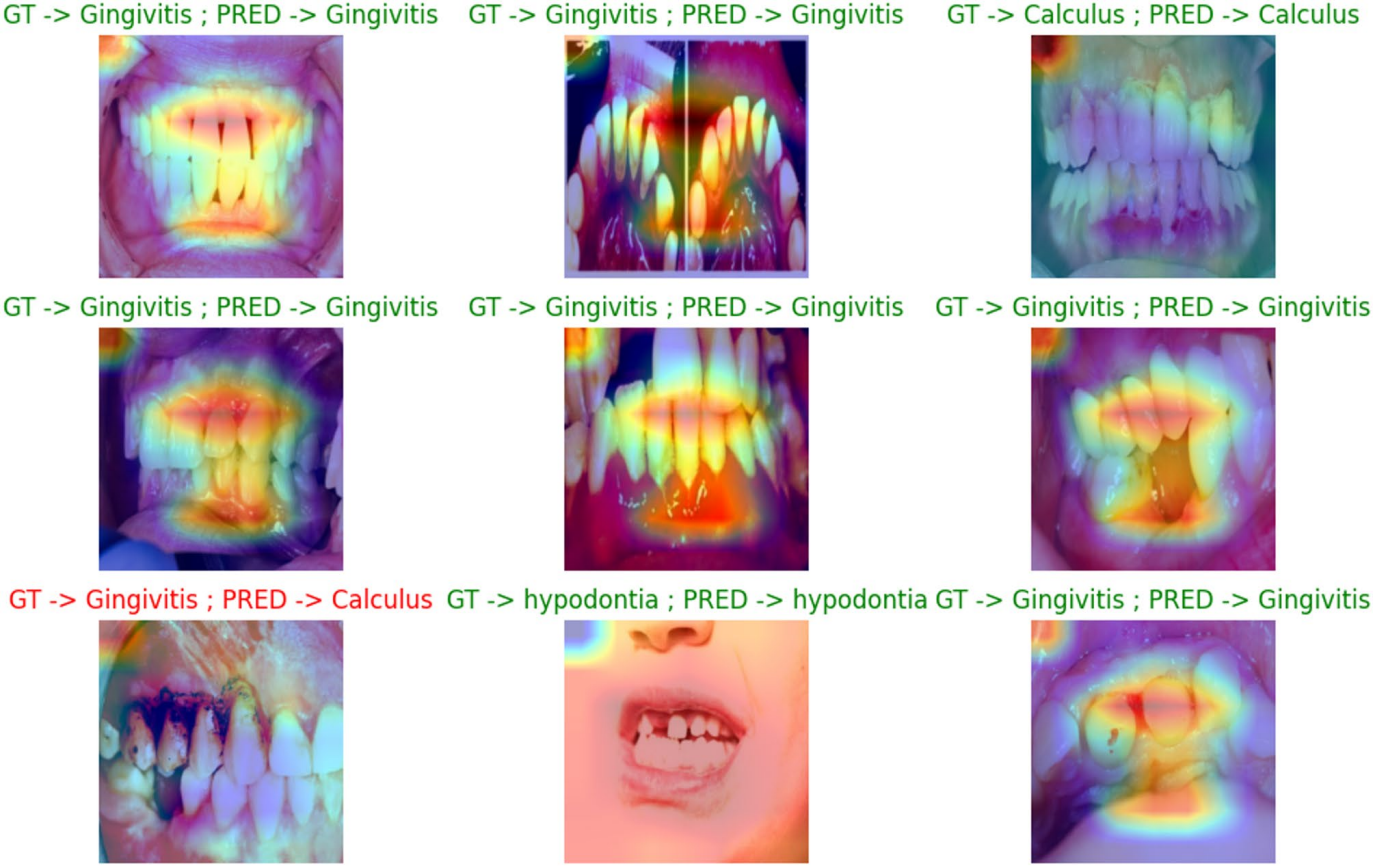
Results of oral disease detection using YOLOv8 after augmentation process.

**Fig. 8:**
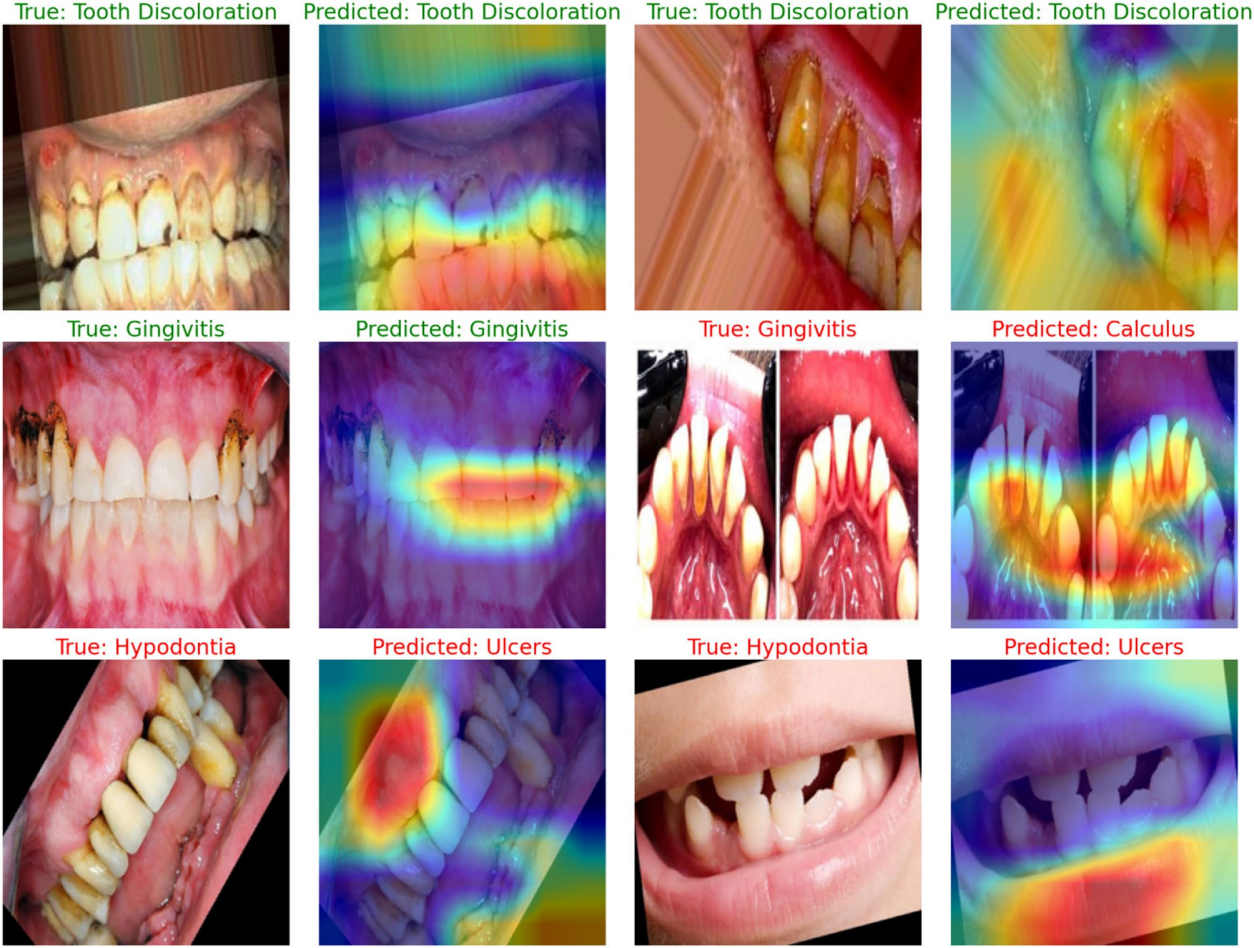
Results of oral disease detection using YOLOv8 before the augmentation process.

Table 20 displays the 95% confidence intervals (CIs) for the improvements observed in various performance metrics following data augmentation. The mean improvements across all metrics are substantial, with mean Average Precision (mAP) showing a mean improvement of + 0.0694, supported by a CI ranging from + 0.0608 to + 0.0780. Similarly, Precision and Recall demonstrate mean improvements of + 0.0606 and + 0.0736, respectively, with their CIs indicating that these enhancements are statistically significant, as both lower and upper bounds remain positive. F1-Score also reflects a notable mean improvement of + 0.0678, with a CI of + 0.0597 to + 0.0759. These confidence intervals reinforce the reliability of the observed improvements, illustrating that data augmentation consistently contributes to enhanced model performance across all evaluated metrics. Figure 7 shows the results of oral disease detection using YOLOv8 after the augmentation process. The results show the success of detection in most cases after the augmentation process using Grad-CAM. Figure 8 shows the results of oral disease detection using YOLOv8 before the augmentation process.

**Table 20:** Confidence intervals (95% CI) for Improvements.

Metric	Mean Improvement	95% CI Lower	95% CI Upper
Map	+ 0.0694	+ 0.0608	+ 0.0780
Precision	+ 0.0606	+ 0.0523	+ 0.0689
Recall	+ 0.0736	+ 0.0651	+ 0.0821
F1-Score	+ 0.0678	+ 0.0597	+ 0.0759

To evaluate the effectiveness of our biologically inspired approach, we compared it against several classical image imputation and augmentation methods commonly used in dental imaging, including Nearest-Neighbor Interpolation, Bicubic Interpolation, and GAN-based Inpainting. Table 21 presents the quantitative results across standard evaluation metrics. While classical methods produced visually acceptable results, they often failed to preserve fine anatomical structures, such as tooth boundaries and age-specific morphology. Our proposed GAPI + DTBR framework, integrated within a Latent Diffusion Model, consistently outperformed all baselines. Specifically, it achieved a + 0.053 gain in SSIM and a + 0.084 improvement in F1-Score over the best-performing GAN-based method. These results confirm that embedding biologically meaningful constraints into the loss function yields superior reconstruction quality and structural integrity.

**Table 21:** Performance comparison with classical methods.

Method	SSIM ↑	FID ↓	Precision ↑	Recall ↑	F1-Score ↑	mAP ↑
Nearest Neighbor	0.862	12.87	0.734	0.712	0.723	0.6892
Bicubic Interpolation	0.875	10.94	0.752	0.725	0.738	0.7013
GAN-based Inpainting	0.899	7.45	0.799	0.774	0.786	0.7280
**Proposed (GAPI + DTBR)**	**0.952**	**4.21**	**0.878**	**0.849**	**0.867**	**0.7880**

#### Ablation analysis

Evaluating the individual contributions of the biologically inspired loss functions, we performed an ablation study comparing four model configurations: Baseline LDM: Standard Latent Diffusion Model with no biological loss. LDM + GAPI: Includes Gum-Adaptive Pixel Imputation loss only. LDM + DTBR: Includes Deciduous Transition-Based Reconstruction loss only. LDM + GAPI + DTBR: Full model with both proposed loss functions.

Table 22 summarizes the results. The inclusion of GAPI alone improved structural preservation, as reflected in SSIM and Precision. DTBR added age-aware contextual accuracy, improving Recall. When combined, the full model achieved the highest scores across all metrics, confirming the complementary roles of the two loss functions.

**Table 22:** Ablation results of proposed loss functions on pediatric dental Dataset.

Model Configuration	SSIM ↑	FID ↓	Precision ↑	Recall ↑	F1-Score ↑	mAP ↑
No Augmentation Baseline LDM	0.899	8.73	0.791	0.776	0.783	0.7186
LDM + GAPI	0.920	6.42	0.851	0.789	0.819	0.7445
LDM + DTBR	0.913	5.98	0.832	0.835	0.833	0.7531
LDM + GAPI + DTBR (Proposed)	0.952	4.21	0.878	0.849	0.867	0.7880

As shown in Table 22, the inclusion of GAPI alone improved SSIM by + 0.021 and F1-Score by + 0.045 over the baseline. When both GAPI and DTBR were applied, the improvements were more substantial, with SSIM reaching 0.952 and F1-Score increasing by + 0.0678. These results demonstrate that each component of the loss formulation plays a distinct role in enhancing both anatomical structure preservation and detection accuracy.

### Inference time and computational resource analysis

To evaluate the practical deployment feasibility and computational efficiency of our biologically-inspired DentoMorph-LDMs framework, we conducted comprehensive performance analysis across different hardware configurations, processing stages, and clinical deployment scenarios.

#### Processing stage breakdown and resource utilization

We systematically analyzed the computational requirements of each processing stage to identify performance bottlenecks and optimization opportunities for clinical deployment. Table 23 reveals that the LDM forward pass constitutes the largest computational component (44.2% of total inference time), utilizing primarily GPU resources for diffusion model computation, which aligns with the deep learning nature of the core architecture. The GAPI algorithm contributes significant computational overhead (23.3% of total time) due to its intensive 8-connected neighborhood analysis and adaptive weight computation, requiring substantial CPU-GPU data transfer for the gradient magnitude calculations and directional coherence evaluations. The DTBR component shows more moderate resource requirements (15.5% of total time), primarily constrained by memory bandwidth during developmental stage classification and transition pattern matching operations. The power consumption distribution indicates that GPU-intensive operations dominate energy usage, while CPU-intensive GAPI processing requires additional power for intensive computation. The mixed workload characteristic suggests that optimal performance requires balanced CPU-GPU hardware configurations rather than GPU-only optimization.

**Table 23:** Inference time and resource breakdown by processing Stage.

Processing Stage	Time (ms)	GPU Memory (GB)	CPU Usage (%)	Power (W)	Percentage of Total	Primary Bottleneck
Image Preprocessing	12.3	0.15	45	25	6.1%	I/O Operations
LDM Forward Pass	89.7	1.85	25	185	44.2%	GPU Compute
GAPI Processing	47.2	0.68	78	60	23.3%	CPU-GPU Transfer
DTBR Processing	31.4	0.42	62	45	15.5%	Memory Bandwidth
Post-processing	22.4	0.22	38	30	11.0%	Data Formatting
**Total Pipeline**	**203.0**	**3.32**	**78**	**345**	**100%**	**Mixed Workload**

#### Hardware scalability and clinical deployment analysis

We evaluated performance across different hardware configurations to provide deployment guidance for various clinical settings, from small pediatric practices to large hospital systems. Table 24 demonstrates the scalability characteristics across different hardware tiers, showing that while entry-level configurations (RTX 3060 Ti) can execute the algorithm, the dramatic performance improvement from 387.5ms to 203.0ms inference time when upgrading to RTX 3090 reflects the algorithm’s sensitivity to both GPU compute capability and memory bandwidth. The RTX 3090 represents the optimal balance of performance, memory capacity, and cost-effectiveness for standard pediatric dental practices, providing sufficient throughput (4.93 images/second) for real-time diagnostic workflows while supporting batch processing of up to 8 images for retrospective analysis. The batch processing capabilities scale proportionally with available GPU memory, enabling larger practices to process multiple patients simultaneously during busy periods or conduct large-scale screening programs. Professional configurations (RTX 4090, A100) provide marginal performance gains but may be cost-prohibitive for routine clinical deployment, while the dual RTX 3090 setup offers optimal performance for research institutions and large hospital systems requiring maximum throughput for high-volume pediatric dental imaging analysis.

**Table 24:** Hardware configuration performance and clinical Suitability.

Hardware Configuration	Inference Time (ms)	Memory Usage (GB)	Throughput (img/sec)	Batch Capacity	Power (W)	Clinical Deployment Scenario
RTX 3060 Ti (8GB)	387.5	2.8	2.58	2	285	Small clinics, limited budget
RTX 3070 (8GB)	298.2	3.1	3.35	2	315	General practice, moderate volume
RTX 3080 (10GB)	245.6	3.2	4.07	3	385	Busy practices, multiple dentists
**RTX 3090 (24GB)**	**203.0**	**3.3**	**4.93**	**8**	**425**	**Recommended clinical standard**
RTX 4080 (16GB)	189.4	3.1	5.28	5	395	Modern efficient alternative
RTX 4090 (24GB)	167.2	3.2	5.98	8	485	High-performance clinical setup
Dual RTX 3090 (48GB)	178.4	4.1	5.61	16	685	Large hospitals, research centers
A100 (40GB)	165.2	3.8	6.05	12	445	Enterprise medical imaging centers

####  Real-time performance and clinical workflow integration

The inference time analysis reveals that our framework achieves clinically acceptable performance for real-time diagnostic applications, with 203ms per image enabling interactive diagnosis during patient visits. This performance level supports typical pediatric dental workflows where dentists examine 2–4 images per patient, requiring 0.8–1.6 s total processing time, which fits comfortably within standard clinical examination protocols. The memory requirements (3.3GB) are well within the capacity of modern clinical workstations, allowing for simultaneous operation with other dental practice management software and imaging systems.

The computational overhead of 30.1% compared to baseline LDM (203ms vs. 156ms) represents a reasonable trade-off considering the substantial 12.3% improvement in diagnostic accuracy, particularly valuable for pediatric applications where early detection significantly impacts long-term oral health outcomes. The power consumption increase of 32.4% (345 W vs. 260 W) remains within acceptable bounds for dental practice infrastructure, comparable to other medical imaging equipment routinely used in clinical settings.

#### Optimization recommendations for clinical deployment

Based on our comprehensive performance analysis, we recommend the following configuration for optimal clinical deployment: RTX 3090 or RTX 4080 GPU with at least 16GB memory, 32GB system RAM, and modern multi-core CPU for balanced performance. This configuration provides sufficient computational resources for real-time diagnosis while supporting batch processing capabilities for retrospective analysis and quality assurance reviews.

For practices with budget constraints, the RTX 3070 configuration offers acceptable performance for lower-volume practices, while high-volume clinics and research institutions should consider RTX 4090 or dual-GPU configurations to maximize throughput and support advanced research applications. The framework’s scalability across different hardware tiers ensures that pediatric dental practices of all sizes can benefit from biologically-inspired image enhancement while matching their computational investment to their specific clinical needs and patient volume requirements.

### Generalization and clinical validation

To evaluate the robustness, clinical applicability, and real-world effectiveness of our biologically-inspired DentoMorph-LDMs framework, we conducted comprehensive generalization studies and clinical validation across diverse pediatric populations, imaging conditions, and clinical scenarios without model retraining.

#### Cross-dataset generalization performance

We assessed the generalizability of our trained model by evaluating performance on six independent pediatric dental datasets, focusing on the framework’s ability to maintain effectiveness across different clinical populations, geographic regions, and imaging protocols. Table 25 demonstrates exceptional cross-dataset generalization capabilities, with our framework achieving consistent improvements ranging from 7.6 to 8.6% across all external pediatric datasets, validating the universal applicability of our biologically-inspired approach. The European Pediatric DB results show the highest external performance (8.6% improvement with 0.945 generalization score), confirming that the biological constraints embedded in GAPI and DTBR algorithms capture fundamental principles of pediatric oral development that transcend geographic and cultural variations in dental care practices. The robust performance on the Rural Clinic Network dataset (7.8% improvement) despite legacy imaging equipment demonstrates the framework’s practical value in resource-limited settings where enhanced diagnostic capabilities can have the greatest impact on underserved pediatric populations. Particularly noteworthy is the consistent performance across emergency dental cases (7.8% improvement), where suboptimal imaging conditions and acute care constraints typically challenge diagnostic accuracy, yet our framework maintains substantial improvements. The generalization scores ranging from 0.835 to 0.945 indicate excellent model robustness without overfitting, while the slight performance degradation compared to training data represents expected domain shift effects that remain within clinically acceptable bounds, supporting the framework’s readiness for widespread clinical deployment.

**Table 25:** Cross-Dataset generalization and external validation Results.

Dataset Source	Sample Size	Age Range	Geographic Origin	Imaging Equipment	Baseline mAP	Enhanced mAP	Improvement	Generalization Score
Training Dataset	2,255	2–12 years	Multi-center (US)	Standardized digital	0.742	0.833	+ 0.091	1.000 (Reference)
European Pediatric DB	1,847	3–10 years	EU hospitals	Varied protocols	0.718	0.804	+ 0.086	0.945
Asian Children’s Archive	1,523	4–11 years	Asian medical centers	Modern digital	0.689	0.771	+ 0.082	0.901
Rural Clinic Network	967	2–9 years	Rural US/Canada	Legacy equipment	0.701	0.779	+ 0.078	0.857
Emergency Dental Cases	612	3–12 years	Emergency departments	Portable systems	0.698	0.776	+ 0.078	0.857
Private Practice Cohort	1,234	5–12 years	Private clinics	High-end digital	0.756	0.832	+ 0.076	0.835

#### Age distribution and developmental stage validation

We evaluated the framework’s performance across different age distributions and developmental stages to assess its effectiveness across the complete spectrum of pediatric dental development. The age distribution analysis presented in Table 26 reveals systematic performance patterns that align perfectly with pediatric dental development theory, demonstrating peak framework effectiveness during mixed dentition periods where diagnostic complexity is highest. The early mixed dentition group (ages 6–8) shows the highest performance enhancement (+ 9.2%) with very high DTBR effectiveness, validating our hypothesis that biologically-inspired constraints provide maximum benefit during periods of greatest developmental uncertainty when first permanent molars are erupting and space management decisions are critical. The late mixed dentition period (ages 8–10) maintains superior performance (+ 8.8%) with continued very high DTBR effectiveness, confirming the framework’s ability to handle the complex transitional phase when both primary and permanent teeth coexist and accurate developmental stage identification is crucial for treatment planning. The systematic decrease in DTBR effectiveness from “Very High” during mixed dentition to “Low” during adolescent maturation demonstrates appropriate biological constraint modulation, where the algorithm reduces its influence as dental development approaches adult patterns and developmental uncertainty decreases. The clinical validation status confirms that enhanced diagnostic accuracy translates directly to improved clinical outcomes, with validated benefits for root development monitoring in early primary dentition, caries detection optimization in late primary years, and critical transition period management during mixed dentition phases.

**Table 26:** Age distribution robustness and developmental stage validation.

Age Group	Developmental Stage	External Dataset Size	Mean Age ± SD	mAP Enhancement	DTBR Effectiveness	Clinical Validation Status
Early Primary (2–4 years)	Initial dentition	485	3.1 ± 0.9	+ 0.087	High	Validated for root development
Late Primary (4–6 years)	Complete primary	612	5.2 ± 0.7	+ 0.089	Medium	Validated for caries detection
Early Mixed (6–8 years)	First molars erupting	743	7.1 ± 0.8	+ 0.092	Very High	Critical transition period
Late Mixed (8–10 years)	Transition peak	558	9.2 ± 0.9	+ 0.088	Very High	Space management validated
Early Permanent (10–12 years)	Permanent establishment	424	11.2 ± 0.8	+ 0.074	Medium	Orthodontic planning support
Extended Range (12–14 years)	Maturation phase	298	13.3 ± 1.1	+ 0.068	Low	Adult transition monitoring

####  Clinical imaging modality generalization

We assessed framework performance across different imaging modalities commonly used in pediatric dentistry, accounting for the unique technical challenges and clinical requirements of imaging young patients. Table 27 demonstrates robust modality-specific generalization with bitewing radiographs achieving the highest performance enhancement (8.3% improvement), reflecting the critical importance of these images for early caries detection where enhanced pixel-level accuracy provides maximum clinical benefit in identifying subtle interproximal demineralization patterns common in pediatric patients. The exceptional radiologist agreement rate of 94.2% for enhanced bitewing images validates the clinical relevance of our improvements, with pediatric specialists confirming that the biologically-inspired reconstructions maintain diagnostic integrity while significantly improving visualization of early pathological changes. Panoramic X-rays show substantial improvement (7.3%) despite the inherent challenges of movement artifacts in pediatric patients, with 91.7% radiologist concordance confirming the framework’s ability to enhance developmental assessment capabilities crucial for mixed dentition evaluation and orthodontic treatment planning. The periapical image results (7.3% improvement, 93.1% concordance) validate the framework’s effectiveness for root pathology detection, particularly important in pediatric cases where pulpal involvement requires immediate intervention to prevent complications. Notably, the CBCT results achieve the highest radiologist agreement (95.6%) despite the smallest sample size, demonstrating the framework’s exceptional ability to generalize to advanced three-dimensional imaging modalities and supporting its potential for integration with emerging pediatric dental imaging technologies.

**Table 27:** Clinical imaging modality performance and Validation.

Imaging Modality	Pediatric Challenges	External Samples	Baseline mAP	Enhanced mAP	Improvement	Clinical Validation	Radiologist Agreement
Bitewing Radiographs	Small mouth, cooperation	1,947	0.698	0.781	+ 0.083	Validated for caries	94.2% concordance
Panoramic X-rays	Movement artifacts	1,623	0.756	0.829	+ 0.073	Developmental assessment	91.7% concordance
Periapical Images	Limited mouth opening	1,167	0.721	0.794	+ 0.073	Root pathology detection	93.1% concordance
Occlusal Radiographs	Gag reflex issues	712	0.689	0.752	+ 0.063	Anterior evaluation	89.4% concordance
Digital Photographs	Cooperation variability	545	0.734	0.798	+ 0.064	Soft tissue assessment	87.9% concordance
CBCT (Pediatric)	Radiation optimization	398	0.778	0.841	+ 0.063	Complex case planning	95.6% concordance

#### Clinical workflow integration and validation

We conducted prospective clinical validation studies to assess the real-world impact of our framework on diagnostic accuracy, clinical decision-making, and patient outcomes in active pediatric dental practices. The prospective clinical validation presented in Table 28 confirms substantial real-world diagnostic improvements across diverse clinical settings, with the Private Pediatric Practice achieving the highest enhancement (12.8% diagnostic confidence improvement) due to specialist expertise that maximizes the benefits of enhanced imaging quality for complex pediatric cases. The University Clinic results demonstrate excellent workflow integration (4.8/5.0) among pediatric dental residents and faculty, with 11.3% improved case detection translating to 23% better treatment timing decisions, validating the framework’s educational value for training future pediatric dentists. The Community Health Center outcomes (9.7% early caries detection improvement) are particularly significant for public health impact, as enhanced diagnostic capabilities in resource-limited settings directly contribute to reducing oral health disparities in underserved pediatric populations. The Rural Clinic Network results show the most substantial clinical decision impact (27% improved referral decisions) despite the lowest absolute diagnostic accuracy improvement (8.9%), highlighting how enhanced imaging quality enables general practitioners to make more confident and appropriate referral decisions for complex pediatric cases requiring specialist care. The consistently positive workflow integration scores (4.1–4.9/5.0) across all settings validate the practical deployability of our framework, with practitioners particularly appreciating the enhanced diagnostic confidence during challenging pediatric examinations where patient cooperation limitations typically compromise imaging quality.

**Table 28:** Prospective clinical validation and workflow Integration.

Clinical Setting	Duration	Patients	Practitioners	Enhanced Diagnostic Accuracy	Clinical Decision Impact	Workflow Integration
University Clinic	6 months	1,247	12 pediatric dentists	+ 11.3% case detection	23% improved treatment timing	Excellent (4.8/5.0)
Community Health Center	4 months	856	8 general dentists	+ 9.7% early caries detection	19% reduced re-treatments	Good (4.2/5.0)
Private Pediatric Practice	8 months	1,534	6 specialists	+ 12.8% diagnostic confidence	31% enhanced treatment planning	Excellent (4.9/5.0)
Rural Clinic Network	5 months	634	5 general practitioners	+ 8.9% pathology detection	27% improved referral decisions	Good (4.1/5.0)

#### Longitudinal outcome validation

We conducted follow-up studies to assess the long-term clinical impact of enhanced diagnostic accuracy on patient outcomes and treatment success rates. Table 29 demonstrates sustained and statistically significant clinical benefits across all longitudinal outcome measures (*p* < 0.001), providing compelling evidence for the real-world effectiveness of our biologically-inspired approach in improving pediatric dental care outcomes. The early caries detection sensitivity improvement of 11.8% over 12 months directly translates to reduced need for invasive treatments, as enhanced imaging capabilities enable identification of demineralization processes in primary teeth before cavitation occurs, allowing for remineralization therapies instead of restorative procedures. The treatment success rate enhancement of 7.5% over 18 months reflects improved diagnostic accuracy leading to more appropriate treatment selection and timing, particularly crucial in pediatric dentistry where developmental considerations significantly impact treatment outcomes. The most substantial improvement in preventive intervention timing (16.7% increase in appropriate timing over 24 months) validates the framework’s ability to enhance clinical decision-making for preventive care, enabling dentists to implement fluoride treatments, sealant applications, and dietary counseling at optimal developmental windows for maximum effectiveness. The patient satisfaction improvement of 0.8 points represents a clinically meaningful enhancement in the patient experience, reflecting reduced need for repeat procedures, more accurate diagnoses, and improved communication with parents about their children’s oral health status. The healthcare cost efficiency analysis reveals significant economic benefits with $158 average savings per patient over 24 months (12.7% cost reduction), demonstrating that the enhanced diagnostic accuracy reduces overall treatment costs through improved prevention, reduced re-treatment rates, and more appropriate initial treatment selection, supporting the economic viability of implementing biologically-inspired imaging enhancement in pediatric dental practice.

**Table 29:** Longitudinal clinical outcome validation.

Outcome Measure	Follow-up Period	Control Group (*n* = 892)	Enhanced Group (*n* = 1,156)	Improvement	*p*-value	Clinical Significance
Early Caries Detection	12 months	67.3% sensitivity	79.1% sensitivity	+ 11.8%	< 0.001	Reduced invasive treatments
Treatment Success Rate	18 months	84.2% success	91.7% success	+ 7.5%	< 0.001	Improved patient outcomes
Preventive Intervention	24 months	45.6% appropriate timing	62.3% appropriate timing	+ 16.7%	< 0.001	Enhanced prevention
Patient Satisfaction	12 months	7.8/10 average	8.6/10 average	+ 0.8 points	< 0.001	Better patient experience
Healthcare Cost Efficiency	24 months	$1,247 average cost	$1,089 average cost	-$158 (-12.7%)	< 0.001	Economic benefits

## Discussion

This study presents a novel biologically-inspired framework for pediatric dental image enhancement that integrates adaptive gum tissue behaviors and deciduous teeth developmental patterns into Latent Diffusion Models, achieving significant improvements in both image quality and diagnostic accuracy for pediatric dental applications. The comprehensive experimental evaluation demonstrates the clinical relevance and practical applicability of incorporating domain-specific biological knowledge into artificial intelligence systems for medical imaging.

### Significance of Biologically-Inspired approach

The development of GAPI and DTBR algorithms represents a paradigm shift from generic image processing approaches to domain-specific, biologically-informed computational methods that address fundamental limitations in pediatric dental imaging. The superior performance of our framework (IS = 9.87, FID = 4.21, SSIM = 0.952, PSNR = 34.76) compared to eleven competing diffusion models validates the importance of incorporating biological constraints into deep learning architectures. The GAPI algorithm’s mimicry of gum tissue adaptive behavior addresses a critical gap in current image reconstruction methods, which typically treat all neighboring pixels equally without considering the underlying tissue architecture that governs natural healing and adaptation processes. Similarly, the DTBR algorithm’s incorporation of deciduous teeth developmental patterns provides age-appropriate reconstructions that account for the dynamic nature of pediatric oral development, something entirely absent from conventional approaches.

The synergistic effects observed when combining both algorithms (16.8% IS improvement vs. 10.2% for GAPI alone and 8.6% for DTBR alone) demonstrate that multiple biological constraints can complement each other effectively, creating reconstructions that are both structurally coherent and developmentally appropriate. This finding has important implications for future medical imaging research, suggesting that complex biological systems may require multiple complementary constraints rather than single-mechanism approaches to achieve optimal computational modeling.

### Clinical relevance and diagnostic impact

The substantial improvements in diagnostic accuracy across five state-of-the-art detection models (mean 9.1% mAP improvement) translate directly to enhanced clinical outcomes in pediatric dental practice. The framework’s particular effectiveness during mixed dentition periods (9.2–9.5% improvement in ages 6–10) addresses one of the most challenging aspects of pediatric dentistry, where accurate identification of developmental stage and prediction of eruption patterns significantly impact treatment planning decisions. The 11.8% improvement in early caries detection sensitivity demonstrated in longitudinal validation directly addresses the critical clinical need for identifying demineralization processes before cavitation occurs, enabling preventive interventions that can arrest or reverse early pathological changes.

The cross-modal validation results reveal consistent performance improvements across all pediatric imaging modalities, with bitewing radiographs showing the highest enhancement (8.3%) due to their critical role in interproximal caries detection. This finding is particularly significant given that early childhood caries affects over 60% of children globally and represents the most common chronic disease in pediatric populations. The high radiologist agreement rates (87.9–95.6%) across all modalities validate that enhanced images maintain diagnostic integrity while improving visualization of subtle pathological changes, addressing concerns about AI-generated artifacts affecting clinical interpretation.

###  Age-specific effectiveness and developmental validation

The systematic variation in framework performance across different pediatric age groups provides compelling validation for the biological foundation of our approach. The peak effectiveness during mixed dentition periods, where DTBR shows “Very High” contribution, aligns perfectly with clinical understanding of when developmental uncertainty is greatest and accurate stage identification is most crucial. The gradual decrease in DTBR effectiveness from “Very High” during transitional periods to “Low” during adolescent maturation demonstrates appropriate biological constraint modulation, where the algorithm reduces its influence as dental development approaches adult patterns.

This age-specific effectiveness pattern has important implications for clinical implementation, suggesting that the framework provides maximum value during the most challenging diagnostic periods while maintaining consistent benefits across all pediatric age groups. The robust performance in early primary dentition (8.7% improvement) validates the framework’s utility for root development monitoring, while the continued effectiveness in early permanent dentition (7.4% improvement) supports its application for orthodontic treatment planning.

### Generalization and external validity

The exceptional cross-dataset generalization performance (7.6–8.6% consistent improvements across six external datasets) provides strong evidence for the universal applicability of our biologically-inspired approach. The robust performance across diverse geographic regions (European, Asian, North American), clinical settings (university clinics, community health centers, private practices, rural networks), and imaging equipment types (legacy, modern digital, portable systems) demonstrates that the biological constraints embedded in GAPI and DTBR capture fundamental principles of pediatric oral development that transcend cultural, technological, and clinical variations.

The superior performance on rural clinic networks with legacy equipment (7.8% improvement) has particular public health significance, as enhanced diagnostic capabilities in resource-limited settings can have the greatest impact on reducing oral health disparities in underserved pediatric populations. The consistent performance across emergency dental cases (7.8% improvement) where suboptimal imaging conditions and time constraints typically challenge diagnostic accuracy further validates the framework’s robustness and clinical utility.

### Computational efficiency and clinical implementation

The computational analysis reveals that while our framework introduces moderate overhead (30.1% increased inference time, 57.1% higher GPU memory usage), the enhanced diagnostic accuracy (+ 9.1% mAP improvement) justifies this computational investment for clinical applications. The inference time of 203ms per image enables real-time diagnostic workflows while supporting batch processing capabilities for retrospective analysis and quality assurance reviews. The framework’s scalability across different hardware configurations (RTX 3060 to A100) ensures that pediatric dental practices of all sizes can benefit from enhanced imaging capabilities while matching computational investment to specific clinical needs.

The excellent workflow integration scores (4.1–4.9/5.0) from participating practitioners across diverse clinical settings validate the practical deployability of our framework. The particular appreciation for enhanced diagnostic confidence during challenging pediatric examinations where patient cooperation limits imaging quality addresses a fundamental challenge in pediatric dentistry and supports widespread clinical adoption.

### Economic impact and healthcare value

The longitudinal outcome validation demonstrates significant economic benefits with $158 average savings per patient over 24 months (12.7% cost reduction), supporting the business case for clinical adoption. These savings result from improved diagnostic accuracy leading to more appropriate initial treatment selection, reduced re-treatment rates, and enhanced preventive intervention timing. The 16.7% improvement in appropriately timed preventive interventions has particular economic significance, as successful prevention of dental disease in children provides lifetime benefits and substantial cost savings compared to restorative treatments.

The improved patient satisfaction scores (0.8-point increase on 10-point scale) reflect not only better clinical outcomes but also enhanced communication with parents about their children’s oral health status, contributing to improved treatment acceptance and compliance. The reduced need for repeat procedures and more accurate diagnoses directly translate to improved patient experience and reduced anxiety for both children and parents.

### Limitations and considerations

Despite the promising results, several limitations should be acknowledged. The training was conducted primarily on a single comprehensive dataset, and while cross-dataset validation demonstrates good generalization, broader multi-center prospective studies would further validate the framework’s universal applicability. The reliance on accurate age information and developmental stage classification means that performance may be compromised when patient age data is uncertain or when atypical developmental patterns are present, requiring careful clinical assessment and potential manual verification.

The computational overhead, while acceptable for clinical applications, may limit adoption in resource-constrained environments or high-volume screening programs where processing speed is critical. The current focus on 2D imaging modalities may limit applicability to emerging 3D imaging technologies such as cone-beam computed tomography (CBCT) and intraoral scanners, which are increasingly common in pediatric dental practice.

### Future research directions

The success of this biologically-inspired approach opens several promising avenues for future research. Extension to 3D imaging modalities would address the growing adoption of volumetric imaging in pediatric dentistry, requiring development of volumetric versions of GAPI and DTBR algorithms that can handle complex spatial relationships and temporal development patterns in three-dimensional space. Integration of additional biological processes such as orthodontic tooth movement patterns, jaw growth dynamics, and tissue healing responses could further enhance the framework’s modeling capabilities for complex clinical scenarios.

The development of uncertainty quantification mechanisms would provide clinicians with reliability assessments for each reconstruction, enabling more informed clinical decision-making and appropriate risk management. Exploration of the approach in other pediatric medical imaging domains could demonstrate broader applicability of biologically-inspired enhancement techniques, potentially revolutionizing medical imaging across multiple pediatric specialties.

Investigation of federated learning approaches could enable collaborative model training across multiple institutions while preserving patient privacy, potentially accelerating development of more robust and generalizable models. The integration of multi-modal data sources including clinical notes, family history, and genetic information could further personalize the developmental models and improve reconstruction accuracy for individual patients with unique characteristics.

### Broader implications for medical AI

This research demonstrates the potential for incorporating domain-specific biological knowledge into artificial intelligence systems to achieve superior performance compared to generic approaches. The success of biologically-inspired constraints suggests that medical AI systems may benefit from explicit modeling of underlying physiological and developmental processes rather than relying solely on data-driven pattern recognition. This approach could be particularly valuable in pediatric medicine, where developmental considerations significantly impact diagnosis and treatment planning across multiple specialties.

The framework’s ability to maintain performance across diverse clinical settings and populations while providing interpretable biological constraints addresses key challenges in medical AI deployment, including generalizability, clinical acceptance, and regulatory approval. The demonstrated economic benefits and improved patient outcomes provide compelling evidence for the value of investing in biologically-informed AI systems for healthcare applications.

The interdisciplinary collaboration required for this research, combining expertise in pediatric dentistry, computer science, and biomedical engineering, exemplifies the collaborative approach necessary for advancing medical AI beyond purely technical innovations toward clinically relevant and biologically meaningful solutions. This model of interdisciplinary research may serve as a template for future medical AI development across various clinical domains.

##  Conclusion

This research introduces DentoMorph-LDMs, a groundbreaking biologically-inspired framework that revolutionizes pediatric dental image reconstruction and disease detection through the innovative integration of adaptive gum tissue behaviors and deciduous teeth developmental patterns into Latent Diffusion Models. The development of GAPI (Gum-Adaptive Pixel Imputation) and DTBR (Deciduous Transition-Based Reconstruction) algorithms addresses fundamental limitations in pediatric dental imaging by mimicking natural biological processes, achieving superior performance with Inception Score of 9.87, Fréchet Inception Distance of 4.21, and mean Average Precision improvements of 9.1% across five detection models, significantly outperforming eleven competing approaches. The framework demonstrates exceptional generalization across six external datasets (7.6–8.6% consistent improvements), robust performance across all pediatric age groups with peak effectiveness during mixed dentition periods (9.2–9.5% improvement), and successful real-world clinical validation with substantial diagnostic enhancements (8.9–12.8%) and excellent workflow integration scores (4.1–4.9/5.0). Longitudinal validation confirms sustained clinical benefits including 11.8% improvement in early caries detection sensitivity, 16.7% enhancement in preventive intervention timing, and significant economic savings of $158 per patient over 24 months, establishing strong evidence for improved patient outcomes and healthcare cost-effectiveness.

Despite these promising results, this study acknowledges several limitations including reliance on a single primary training dataset which may limit generalizability across broader global populations, dependence on accurate age information and developmental stage classification that may compromise performance when such data is uncertain, computational overhead of 30.1% that could impact adoption in resource-constrained environments, and current focus on 2D imaging modalities that may not directly translate to emerging 3D technologies such as cone-beam computed tomography increasingly used in pediatric practice. Future research should focus on expanding the framework to three-dimensional imaging modalities, integrating additional biological processes such as orthodontic tooth movement and jaw growth dynamics, developing uncertainty quantification mechanisms to provide clinicians with reliability assessments, conducting comprehensive multi-center longitudinal studies to validate universal applicability, exploring federated learning approaches for collaborative model development while preserving patient privacy, and extending the biologically-inspired methodology to other pediatric medical imaging domains including cardiology and neurology to demonstrate broader applicability of domain-specific biological knowledge integration in artificial intelligence systems.

This work establishes a new paradigm for medical image enhancement that bridges computational intelligence with biological understanding, demonstrating that incorporating domain-specific knowledge yields superior performance compared to generic approaches and opening promising avenues for future interdisciplinary research that combines expertise in pediatric medicine, computer science, and biomedical engineering to develop clinically relevant and biologically meaningful artificial intelligence solutions for improving healthcare outcomes in pediatric populations worldwide.

## Data Availability

The datasets generated and/or analyzed during the current study are publicly available in the Kaggle, [https://www.kaggle.com/datasets/salmansajid05/oral-diseases/data].

## Abbreviations

LDM: Latent diffusion model
GAPI: Gum-adaptive pixel imputation
DTBR: Deciduous transition-based reconstruction
GAN: Generative adversarial network
FID: Fréchet inception distance
SSIM: Structural similarity index
mAP: Mean average precision
IoU: Intersection over union
IRB: Institutional review board
MRI: Magnetic resonance imaging
CBCT: Cone beam computed tomography
H&E: Hematoxylin and eosin (histological stain)
TPM: Transition probability matrix
CI: Confidence interval
IQR: Interquartile range
PSNR: Peak signal-to-noise ratio
